# Rational design of a near-infrared fluorescent probe for monitoring butyrylcholinesterase activity and its application in development of inhibitors

**DOI:** 10.3389/fbioe.2024.1387146

**Published:** 2024-04-04

**Authors:** Hao Li, Xiao-Dong Li, Chao-Hua Yan, Zhen-Hua Ni, Mu-Han Lü, Li-Wei Zou, Ling Yang

**Affiliations:** ^1^ Shanghai Frontiers Science Center of TCM Chemical Biology, Institute of Interdisciplinary Integrative Medicine Research, Shanghai University of Traditional Chinese Medicine, Shanghai, China; ^2^ Department of Gastroenterology, The Affiliated Hospital of Southwest Medical University, Luzhou, China

**Keywords:** Butyrylcholinesterase, near-infrared, fluorescent probe, high-throughput screening, flavonoids

## Abstract

Butyrylcholinesterase (BChE) is widely expressed in multiple tissues and has a vital role in several key human disorders, such as Alzheimer’s disease and tumorigenesis. However, the role of BChE in human disorders has not been investigated. Thus, to quantitatively detect and visualize dynamical variations in BChE activity is essential for exploring the biological roles of BChE in the progression of a number of human disorders. Herein, based on the substrate characteristics of BChE, we customized and synthesized three near-infrared (NIR) fluorescent probe substrates with cyanine-skeleton, and finally selected a NIR fluorescence probe substrate named CYBA. The CYBA demonstrated a significant increase in fluorescence when interacting with BChE, but mainly avoided AChE. Upon the addition of BChE, CYBA could be specifically hydrolyzed to TBO, resulting in a significant NIR fluorescence signal enhancement at 710 nm. Systematic evaluation revealed that CYBA exhibited exceptional chemical stability in complex biosamples and possessed remarkable selectivity and sensitivity towards BChE. Moreover, CYBA was successfully applied for real-time imaging of endogenous BChE activity in two types of nerve-related living cells. Additionally, CYBA demonstrated exceptional stability in the detection of complex biological samples in plasma recovery studies (97.51%–104.01%). Furthermore, CYBA was used to construct a high-throughput screening (HTS) method for BChE inhibitors using human plasma as the enzyme source. We evaluated inhibitory effects of a series of natural products and four flavonoids were identified as potent inhibitors of BChE. Collectively, CYBA can serve as a practical tool to track the changes of BChE activity in complicated biological environments due to its excellent capabilities.

## 1 Introduction

Butyrylcholinesterase (BChE) is a human cholinesterase widely distributed in plasma, liver, muscle tissues, and brain tissues (Santarpia et al., 2013; Lockridge, 2015; Masson et al., 2023). The structure and function of BChE and acetylcholinesterase (AChE) are similar, and their research dates back to 1932 (Stedman et al., 1932). AChE is primarily involved in the hydrolysis of acetylcholine (ACh), whereas BChE plays a key role in the hydrolysis of various choline conjugates containing ACh, butyrylcholine and succinylcholine, as well as numerous drugs such as cocaine, heroin, and aspirin (Darvesh et al., 2003; Kolarich et al., 2008; Long and Cravatt, 2011). As opposed to AChE, the exact biological role of BChE is not well-known. It is reported that BChE participates in various human diseases, such as cardiovascular disease (Goliasch et al., 2012), diabetes (Sridhar et al., 2010), hypercholesterolemia (Wang et al., 2022) and neurodegenerative disorders (Jasiecki et al., 2021; Pathak and Kabra, 2024). Studies have shown that the therapeutic strategy of inhibiting BChE activity can effectively improve cognitive function in patients with Alzheimer’s disease (AD) (Macdonald et al., 2017; De Candia et al., 2023). Moreover, recent studies have also shown BChE is overexpressed in numerous tumorigenesis, such as breast cancer (Boberg et al., 2013; Kumar et al., 2017), colorectal carcinoma (Morera Ocón et al., 2007), oral squamous cell carcinoma (Jaiswal and Jaiswal, 2020), prostate cancer (Gu et al., 2018) and lung carcinoma (Santarpia et al., 2013; Shin et al., 2017). Thus, to quantitatively detect and visualize the dynamical changes in BChE activity in living systems under various situations will be essential for exploring the biological roles of BChE in the development of a number of human diseases.

Currently, the prevailing technique for determining BChE activity is Ellman’s method, which relies on the hydrolysis of butyrylthiocholine to product thiocholine (Ellman et al., 1961). This is also the most popular method for screening BChE inhibitors (Cetin et al., 2021a; Cetin et al., 2021b). Thiocholine reacts with 5,5′-dithio-bis-2-nitrobenoic acid (DTNB), a thiol-capturing Ellman’s reagent and forms a yellow product could be measured at 412 nm (Pohanka, 2014; de Almeida et al., 2022). However, it is not a direct method. And it easily induces “false positive”as various compounds containing sulfhydryl groups also could react with DTNB (Dingova et al., 2014). Meanwhile, it also could not track the changes in BChE activity in living systems. Furthermore, other approaches for detecting BChE include electrochemical, colorimetric techniques, and ELISA assays, while these approaches are more expensive and not sufficiently sensitive (Orhan et al., 2017; Ozcelikay et al., 2019; Xu et al., 2019). By contrast, fluorescence probe is quite attractive to monitor BChE activity as it is a direct method and possess high selectivity and sensitivity (Guo et al., 2023; Dirak et al., 2024; Jiang et al., 2024). Thus, developing a precise and specific fluorescent probe for assessing BChE activity and screening inhibitors is very desirable and a critical issue.

Recently, several fluorescent probes have been reported for the detection of BChE. However, developing specific substrates for BChE activity still remains a great challenge due to the interference from AChE, which is co-existed with BChE in the biological sample (Ma et al., 2020). Moreover, most probes are still restricted to monitoring the level of enzyme activity in cells, as the use of short-wavelength probes impedes their further application *in vivo* (Yu et al., 2022; Zhang et al., 2022). And short Stoke shifts and poor sensitivity limit the further applications of BChE probes (Supplementary Table S1). It is a general accepted that fluorescence with a longer wavelength usually exhibits a low external interference, so the attempt of designing probes with near-infrared (NIR) emission is appearing (Reja et al., 2020; Xu et al., 2023). It is reported that cyanine dyes exhibit remarkable optical characteristics as classic NIR fluorophores (Escobedo et al., 2010). In particular, cyanine 7 (Cy7) provides steady emission in the long-wavelength region (650–950 nm), which is within the bio-transparent window. This could provide a positive signal-to-noise ratio and allows for efficient identification of target substances in biological systems by effectively permeating the tissues. Unfortunately, until now, only two Cy7-based BChE fluorescent probes have been developed. The first probe performed in an on-off mode, while the other designed in a ratiometric sensing mode (Yang et al., 2017; Yuan et al., 2023). However, the advancement of off-on BChE probes is currently in a preliminary phase. The off-on BChE probe utilizing Cy7 as a basic skeleton still remain challenging due to the drawback of complete quenching of fluorescence. In 2011, Shabat. et al. proposed a donor-dual-acceptor π-electron cyanine skeleton (QCy7, also termed as TBO) for generation of novel class of Turn-ON NIR probes (Karton-Lifshin et al., 2011). Due to the simple synthesis technique, unique turn-on option, NIR emission, and larger Stokes-shift, TBO has garnered much of attention (Zhang et al., 2019; 2022; Chai et al., 2023). Following that, numerous TBO-based probes were developed for non-invasive NIR imaging of key biological analytes.

Inspired by the outstanding features of the TBO strategy, we reported the construction of a series of easily synthesized NIR probes for the detection of BChE activity. Among these modifications, CYBA exhibited the optimal combination in terms of stability, specificity, and reactivity toward BChE. Subsequently, CYBA was successfully applied to cellular imaging. Moreover, the fluorescence analysis method based on CYBA allowed for rapid and efficient screening of inhibitors. Four excellent BChE inhibitors were identified from natural products derived from 94 traditional Chinese medicines.

## 2 Materials and methods

### 2.1 Materials and instruments

Recombinant enzymes including fibroblast activation protein (FAP), dipeptidyl peptidase 4 (DPP4), DPP9, AChE, BChE, human pancreatic lipase (hPL), Prolyl oligopeptidase (PREP), Thrombin, bile salt hydrolase (BSH), Cathepsin A (CTSA), Notum were all obtained from Sigma-Aldrich (St. Louis, MO, United States). Human plasma was purchased from Research Institute for Shanghai SCHBIO (Shanghai, China). Amino acids, including Glycine (Gly), Serine (Ser), Valine (Val), arginine (Arg), Alanine (Ala), Asparagine (Asn), Phenylalanine (Phe), Glutamine (Glu), Tyrosine (Tyr), lysine (Lys), Leucine (Leu), Isoleucine (Ile), Tryptophan (Trp), Histidine (His), Glutamine (Gln), Proline (Pro), Threonine (Thr), Cysteine (Cys) and metal ions, containing Ni^2+^, Fe^3+^, Ca^2+^, Fe^2+^, K^+^, NH^+^, Zn^2+^, Cu^2+^, SO_4_^2−^, Cl^−^, Na^+^ were obtained from Sinopharm Chemical Reagent Co., Ltd. (Shanghai, China). Tacrine (a known selective ChE inhibitor), Bis(4-nitrophenyl) phosphate (BNPP, specific and broad-spectrum carboxylase inhibitor), Rivastigmine, Donepezil (specific and noncompetitive AChE inhibitor) and Orlistat (a known inhibitor of human pancreatic lipase) were purchased from Bide Pharmatech Ltd. (Shanghai, China). Dimethyl sulfoxide (DMSO) was purchased from Tedia (Ohio, United States) as a stock solution for dissolving all compounds. Cell Counting Kit-8 (CCK-8) was purchased from Dalian Meilun Biotechnology Co., Ltd. (Dalian, China). The 96 native compounds were purchased from Shanghai yuanye Bio-Technology Co., Ltd. (Shanghai, China).

Ultra-purified water was obtained from a Milli-Q water purification system (Millipore, MA, United States). ^1^H NMR and ^13^C NMR spectra were recorded on the Bruker Avance III 400 MHz spectrometers in DMSO-d6. High resolution mass spectrometry (HRMS) was performed with the UHPLC-Q-Exactive Orbitrap system (Thermo Fisher Scientific Inc., Grand Island, NY, United States). The fluorescence tests were analyzed on a multimode enzyme spectrometer (SpectraMax iD3, Molecular Devices, San Jose, CA, United States).

### 2.2 Synthesis

The detailed synthesis and structural characterization of probes are described in the Supplementary Data S1.

### 2.3 Screening of the probes

AChE or BChE (1 U/mL) and PBS buffer (100mM, pH 7.4) were preincubated at 37°C for 3 min. Subsequently, each probe (final concentration 20 μM) was added and incubated for 30 min, and the fluorescence intensity was measured at a wavelength of 710 nm (λ_ex_ = 610 nm) for CYBA and CYNA, and at a wavelength of 720 nm (λ_ex_ = 630 nm) for CUBA.

### 2.4 Structural characterization of metabolites

Substrate (CYBA or CUBA) and PBS buffer (100mM, pH 7.4) were incubated with BChE for 30 min at 37°C. Then cold acetonitrile was added into the incubations to terminate the reaction. After centrifugation, a preparative HPLC system equipped with a reversed-phase column was used to purify the supernatant. The purified metabolites (TBO and TBBO) were dissolved in dimethylsulfoxide-d_6_ for structural analysis using Bruker 400 NMR spectrometer, with a ^1^H NMR (600 MHz) and ^13^C NMR (150 MHz) recording. Meanwhile, the CYBA metabolites were centrifuged at 20,000 g for 15 min at 4 °C. Subsequently, the supernatant metabolites were all characterized by UHPLC-MS/MS.

### 2.5 Fluorescence response of CYBA toward BChE

The sensing capability of CYBA was performed in the standard incubation system containing PBS buffer (100mM, pH 7.4), appropriate amount of BChE and CYBA, with a final incubation volume of 100 μL. After incubating at 37°C for a series of time, 100 μL cold ethanol (or other solvent) was added to terminate the reaction and detected by microplate reader.

### 2.6 Selectivity and stability assays of CYBA toward BChE

The specificity assays of CYBA (20 μM) were incubated separately with different hydrolases (1 U/mL): FAP, DPP-IV, DPP-IX, AChE, BChE, hPL, PREP, Thrombin, BSH, CTSA, Notum. After a 30 min incubation, the fluorescence intensity was measured. The selectivity profiles of CYBA (20 μM) were performed in the presence of different endogenous amino acids (Gly, Ser, Val, Arg, Ala, Asn, Phe, Glu, Tyr, Lys, Leu, Ile, Trp, His, Gln, Pro, Thr, Cys) or common metal ions (Ni^2+^, Fe^3+^, Ca^2+^, Fe^2+^, K^+^, NH^+^, Zn^2+^, Cu^2+^, SO_4_^2−^, Cl^−^, Na^+^). They were co-incubated with BChE (1 U/mL) at 37°C for 30 min and analyzed the fluorescence change to evaluate the stability of the probe.

For the chemical inhibition, different inhibitors (Tacrine, Bis(4-nitrophenyl) phosphate, Rivastigmine, Donepezil and Orlistat) were preincubated with BChE or plasma at 37°C for 3 min. Then CYBA was added to the reaction mixture for 30 min and the fluorescence intensity was measured. The residual activity was calculated by comparing with the control group (PBS instead of inhibitor solvent).

### 2.7 Enzymatic kinetics of BChE-mediated hydrolysis of CYBA

Enzyme kinetic parameters of BChE were evaluated by the CYBA. After a 3-min preincubation of the BChE (1 U/mL) and PBS buffer (100mM, pH 7.4) at 37°C, a desired concentration of CYBA (0.1–5 μM) was added to initiate the reaction. After 30 min incubation, reaction was terminated by adding equal volume of cold ethanol. And then, the 200 µL reaction mixture was pipetted into a 96-well plate, and the fluorescence intensity was measured at 610 nm and emission wavelength 710 nm. Kinetic parameters (*Km* and *Vmax*) were determined by nonlinear regression analysis using the Michaelis-Menten equation.

$$V=\frac{Vmax\times \left[S\right]}{Km+\left[S\right]}$$

where *Vmax* is the reaction speed when the enzyme is saturated with the substrate; [S] is the concentration of the substrate; *Km* is the Michaelis constant.

### 2.8 Limit of detection (LOD) and limit of quantitation (LOQ)

The limit of detection (LOD) and limit of quantitation (LOQ) was calculated by preparing the different concentrations of BChE and using the following equation.

$$\text{LOD}=\frac{3\sigma }{\kappa }$$

$$\text{LOQ}=\frac{10\sigma }{\kappa }$$

Where σ represents the standard deviation of blank measurement, and κ is the slope of the equation for BChE concentration and fluorescence intensity.

### 2.9 Cell culture and cytotoxicity assays

The HT22 cells (mouse hippocampal neuronal cell line, ATCC, Manassas, United States) and U87MG cells (human malignant glioblastoma cells, ATCC, Manassas, United States) were cultured in Dulbecco’s modified Eagle’s medium (DMEM), which was supplemented with 10% FBS (WelGene, Daegu, Korea). The cells were incubated at a temperature of 37°C under a humidified atmosphere containing 5% CO_2_. The cells were subsequently cultivated on 96-well culture plates, with a density of 1 × 10^4^ cells per well. After 24 h of incubation, cells were treated with various doses of CYBA f**o**r 24 h before being cultured with 10% CCK-8 solution for 2 h. Afterwards, the optical density values were assessed at a wavelength of 450 nm.

### 2.10 Bioimaging of BChE in living cells

The cells were placed in glass-bottom cell culture dishes at a concentration of 3 × 10^5^ cells per well, with each well containing 1.0 mL of culture medium (NEST, 15 mm). The cells were pretreated with or without inhibitors (50 μM tacrine) for 1 hour after being washed three times with PBS to eliminate any leftover serum. Then, the cells were treated with CYBA at a concentration of 10 μM for a duration of 60 min, followed by incubation with Hoechst 33342 at a concentration of 10 μM for 10 min at 37 °C. Subsequently, fluorescence images were captured using a fluorescent microscope manufactured by Echo-lab Revolve (Echo, United States). Images were obtained using the DAPI and Cy5 channel. The fluorescence image data were analyzed by quantifying the fluorescence intensity of individual cells in the images using ImageJ software.

### 2.11 Molecular docking simulations

The AChE (PDB ID: 1B41) and BChE crystal structures (PDB ID: 1P0I) were obtained from the PDB website (http://www.rcsb.org/pdb). First, the water and small molecules in the protein crystal were eliminated using PyMOL 2.3.0. Then, the protein structure was loaded into AutoDocktools (v1.5.6) for processes such as hydrogenation, charge computation, and charge distribution. The docking of CYBA into AChE or BChE was performed using AutoDock Vina (version 1.2.3). A grid box measuring 75 × 75 × 75 Å^3^ with a spacing of 0.375 Å was created to enclose the catalytic pocket. Finally, PyMOL 2.3.0 and Discovery Studio (version 19.1.0) were used to determine the interaction mode of the docking results.

### 2.12 High-throughput screening of BChE inhibitors in natural compounds

The BChE inhibitory effects of natural compounds were detected by using plasma as the enzyme source and CYBA as the fluorogenic substrate. To quickly determine the inhibitory effects of 96 compounds, CYBA was used as the fluorogenic substrate of BChE at 37°C for 30 min. Briefly, plasma (2 μL), PBS buffer (100mM, pH 7.4) and compounds (10 μM) were preincubated at 37°C for 3 min. Next, CYBA (5 μM) was added to the systems and co-incubated at 37°C for 30 min. Then equal volume cold ethanol was added to terminate reaction, and fluorescence intensity was measured by microplate reader (λex = 610 nm, λem = 710 nm). The remaining activity was calculated by comparing with the control group (without compounds). IC_50_ was evaluated by non-linear regression, and the data were shown as mean ± SD.

## 3 Results and discussion

### 3.1 Design and synthesis of NIR fluorescent probes

**SCHEME 1:**
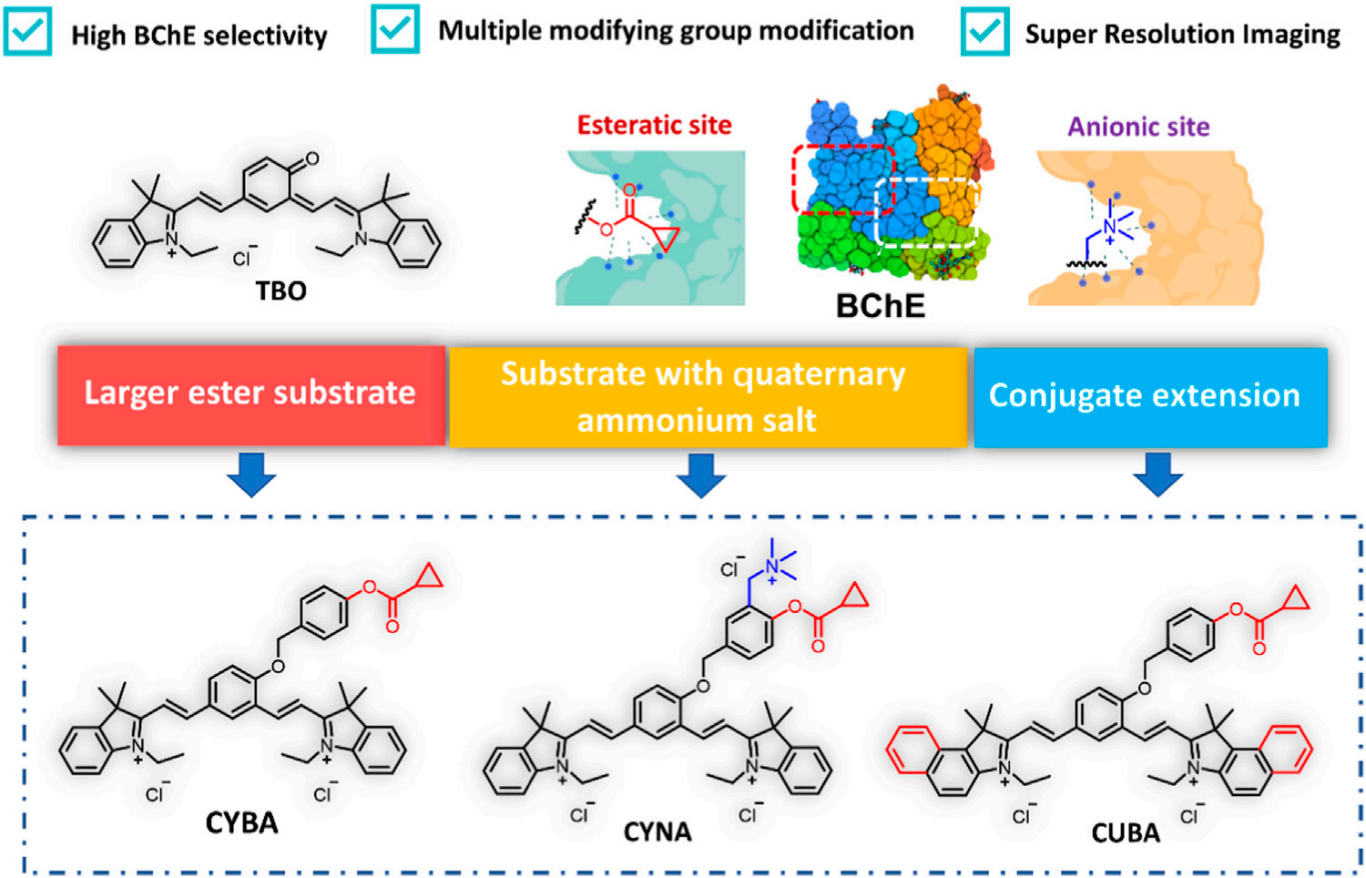
Design of BChE near infrared fluorescence probes. CYBA: 2,2'-((1E, 1′E)-(4-((4-((cyclopropanecarbonyl) oxy) benzyl) oxy)- 1,3-phenylene) bis(ethene-2,1-diyl)) bis(1-ethyl-3,3-dimethyl- 3H-indol-1-ium) chloride, CYNA: 2,2'-((1E, 1′E)-(4-((4-((cyclopropanecarbonyl)oxy)-3-((trimethylammonio) methyl) benzyl) oxy)- 1,3-phenylene) bis(ethene-2,1-diyl)) bis(1-ethyl-3,3-dimethyl-3H-indol-1-ium) chloride, CUBA: 2,2'-((1E, 1′E)-(4-((4-((cyclopropanecarbonyl)oxy) benzyl)oxy)- 1,3-phenylene) bis (ethene-2,1-diyl)) bis (3-ethyl-1,1-dimethyl-1H-benzo [e]indol-3-ium) chloride.

For the purpose of gaining a selective probe for BChE with NIR emission to be applied in biological imaging, we selected cyanine as basic skeleton for developing BChE substrates with long emission wavelengths and excellent photophysical properties. Firstly, we introduced cyclopropyl group as a specific recognition group to construct a specific BChE probe (Yang et al., 2017; Liu et al., 2018). Secondly, considering that the catalytic center of BChE, Ser198-His438-Glu325, is located at the deep parts of the catalytic cavity, 4-hydroxybenzyl alcohol was adopted as the linker to reduce the distance between the recognition group and the catalytic site. Meanwhile, with the elimination of 4-hydroxybenzyl alcohol, intramolecular charge rearrangement could occur, then restore the D-π-A structure and exhibit fluorescence. Thirdly, it is reported an approach to design specific substrates for AChE detection and eliminate the interference from carboxylesterases (CES) by introducing an alkylated ammonium group (Wu et al., 2020). With this in mind, a probe was designed by combining an alkylated ammonium group and a cyclopropyl group to detect BChE while eliminating interference from CES and AChE. Lastly, we constructed a new probe by expanding the conjugated system of the CYBA, with the long emission wavelength to be applied in live cell imaging (Scheme 1). The detailed syntheses, characterization, and photophysical properties of all the compounds were given in the Supplementary Material S1.

### 3.2 Optimization of specific BChE probe

**FIGURE 1:**
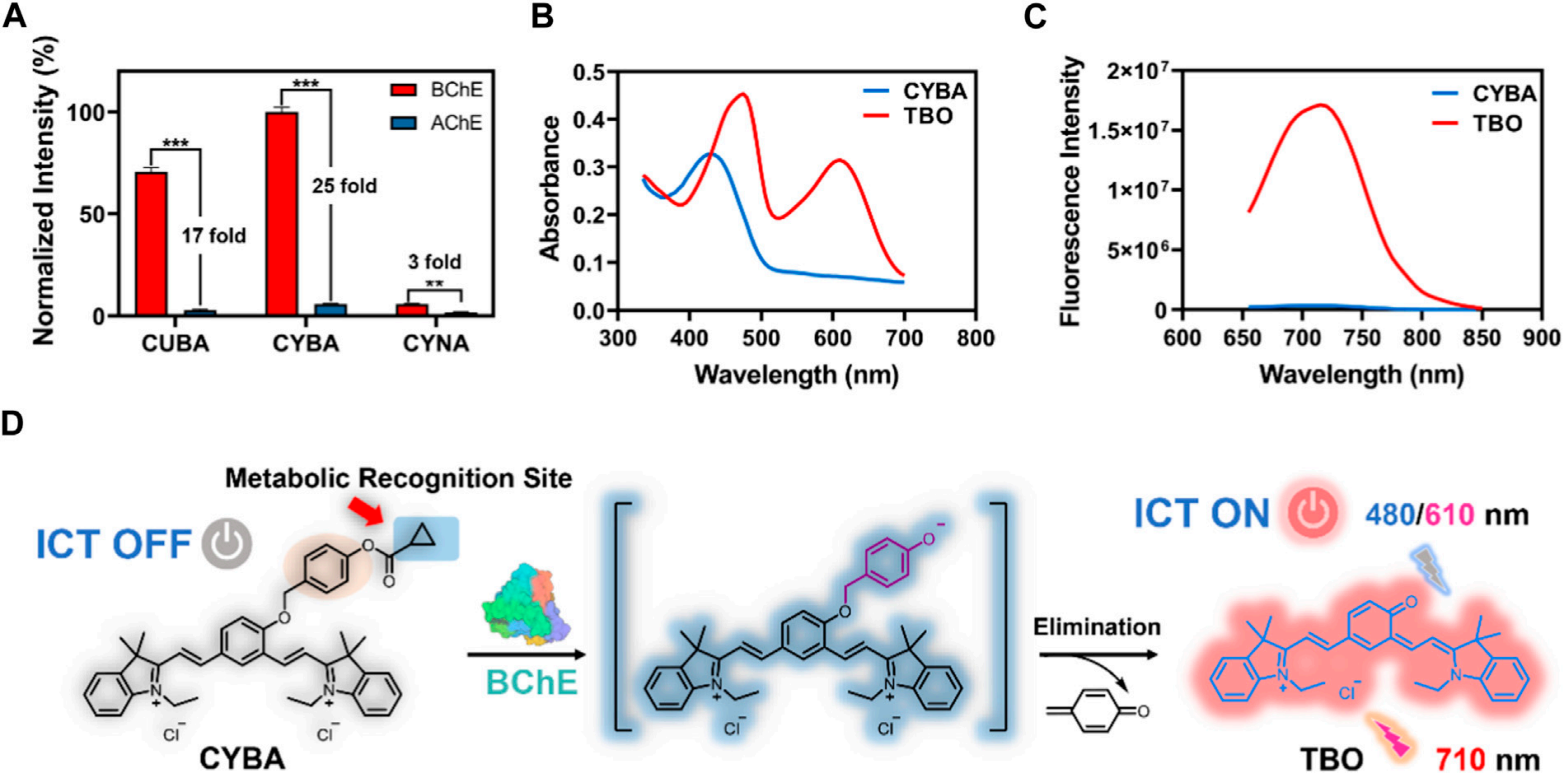
**(A)** Normalized fluorescence intensity of three probes (10 μM) toward BChE and AChE (1 U/mL) at 37^o^C for 30 min in PBS buffer (pH 7.4), respectively. The results are expressed as the mean ± SD (n = 3). The absorption spectra **(B)** and fluorescence spectra **(C)** of CYBA and TBO (20 μM) in PBS–ethanol (v/v = 1:1, pH 7.4). **(D)** Sensing mechanism of CYBA for detecting BChE. ICT: Intramolecular Charge Transfer. **p < 0.01, ***p < 0.001.

We first investigated the reaction rates and specificity of the probes (CYBA, CYNA, CUBA) towards AChE and BChE. Intriguingly, CYBA and CUBA displayed high turnover rates and over 17-fold specificity for BChE over AChE, while CYNA only exhibited weak fluorescence intensity and 3-fold specificity (Figure 1A). And we also found that the inclusion of its quaternary ammonium salt is not only not a beneficial effect, but on the contrary, it also plays a role in hindering the recognition, so the strategy of recognizing the group in the parent nucleus based on TBO with the quaternary ammonium salt needs to be further improved. Subsequently, the products of CYBA and CUBA were identified as TBO and TBBO, which were characterized by ^1^H NMR, ^13^C NMR and HRMS in the Supplementary Material S1. Further experiments showed that the emission wavelength of TBBO was 25 nm longer than that of TBO, which suggesting that the strategy of expanding the conjugated system of the CYBA was useful (Supplementary Figure S1). Regrettably, the fluorescence intensity of CUBA and TBBO was reduced by 38% and 54% with the 60-min incubation at 37 °C (Supplementary Figure S2). CYBA and TBO were stable under the 60-min incubation. Thus, CYBA was chosen as a candidate for subsequent studies.

CYBA itself exhibited an absorption maximum at 430 nm, while the hydrolyzed product TBO showed a dual-band absorption at 480 and 610 nm (Figure 1B). In order to avoid the interference of the substrate and solvent matrix, the 610 nm was chosen as the excitation wavelength of TBO. The maximum emission of TBO is 710 nm, while CYBA exhibits negligible emission at 710 nm (Figure 1C). When the optimization of assay conditions was carried out, it was found that TBO had the strongest fluorescence intensity in the system of PBS-ethanol (v:v = 1:1), and thus this system was used for the product fluorescence assay in the subsequent tests (Supplementary Figure S3). As shown in Supplementary Figure S4, the fluorescent intensity of CYBA was consistent at various pH values, suggesting that this compound was stable over a wide range of pH. TBO could show bright fluorescent signals (pH > 5) and it could serve as a promising probe under physical conditionals. The results indicated that CYBA is capable of functioning effectively under normal physiological settings.

Notably, the fluorescence intensity enhancement was attributed to the hydrolysis of CYBA, which was catalyzed by BChE. To confirm that the fluorescence quenching phenomenon is the result of hydrolysis of CYBA by BChE, we carried out HPLC analysis. As shown in Supplementary Figure S5, CYBA was hydrolyzed after incubation with BChE, and a single metabolite (TBO) was generated. As shown in Figure 1D, CYBA exhibited a polymethine-like structure and utilized a nonfluorescent acceptor-π-acceptor (A-π-A) conjugate formed by the introduction of two electron-withdrawing indolium groups and a recognition moiety. The recognition moiety of CYBA can undergo hydrolytically hydrolyzed by BChE *via* a deprotonation-driven π-electron rearrangement-driven sequential addition-elimination mechanism. In contrast to the initial configuration of CYBA, the donor-π-acceptor (D-π-A) construct facilitates the release of the deprotonated form of TBO, which emits a robust NIR fluorescence signal through an enhanced intramolecular charge transfer (ICT) mechanism.

### 3.3 Characterization of CYBA *in vitro*

**FIGURE 2:**
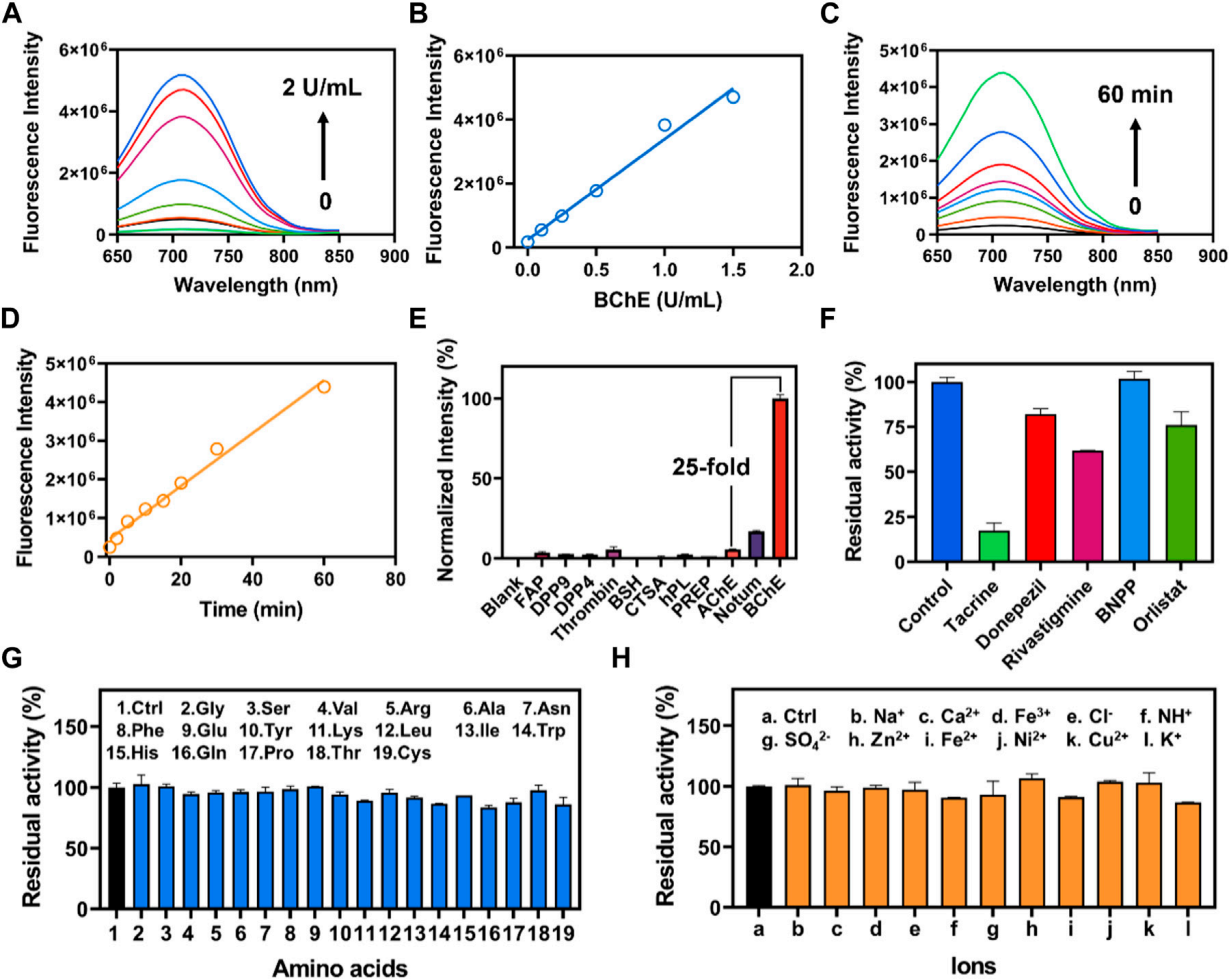
**(A)** The concentration-dependent fluorescence response of CYBA (20 μM) toward BChE (0–2 U/mL) for 30 min at 37 ^o^C in PBS buffer (pH 7.4). **(B)** Linear relationship between fluorescence intensity and BChE concentration (0–2 U/mL). **(C)** The time-dependent fluorescence of CYBA (20 μM) in the presence of BChE (1 U/mL) at 37°C in PBS buffer (pH 7.4). **(D)** Linear relationship between fluorescence intensity and reaction time (0–60 min). **(E)** The selectivity profile of CYBA (5 μM) towards various hydrolases was assessed for 30 min at 37°C in PBS buffer (pH 7.4). **(F)** Chemical inhibition of different inhibitors (10 μM) towards CYBA (5 μM) metabolism. Anti-interference evaluation of CYBA in the presence of various amino acids **(G)** and ions **(H)**. The results are presented as the mean ± SD (n = 3). λ_ex_/λ_em_ = 610/710 nm.

The fluorescence response of CYBA was gradually increased with the addition of various concentration BChE (Figure 2A). The results showed that good linearity relationships (R^2^ = 0.98, *p* < 0.0001) between the fluorescence intensity and enzyme concentrations in the range of 0–1.5 U/mL (Figure 2B). And the detection limit (LOD) and limit of quantitation (LOQ) were evaluated as 0.009 U/mL and 0.03 U/mL, respectively. The time-dependent fluorescence spectra change of CYBA with BChE were further obtained, and the results showed that the fluorescence response exhibited a good linear relationship (R^2^ = 0.98, *p* < 0.001) over 60 min (Figures 2C,D). The fluorescence-based assay demonstrated great reactivity and outstanding selectivity, suggesting that CYBA could be a useful tool for measuring BChE enzyme activities in biological material.

In order to accurately measure the activity of BChE in complex and varied biological samples, it is essential to evaluate both the selectivity and the ability to prevent interference. Hence, we evaluated the specificity and resistance to interference of CYBA. The incubation of CYBA with various common hydrolases was conducted to carry out isoform screening. The results demonstrated the remarkable specificity of CYBA towards BChE, since only BChE could cause a noticeable fluorescence amplification. Conversely, certain human esterases and other proteins that possess hydrolytic activity hardly trigger significantly cause an increase in fluorescence at around 710 nm (Figure 2E). As shown in Figure 2F, tacrine exhibits potent inhibition against BChE-mediated CYBA hydrolysis, while BNPP (a specific inhibitor of carboxylesterases) shows no inhibition. Donepezil and rivastigmine, as a strong inhibitor of AChE and a weak inhibitor of BChE, shows little inhibition on the hydrolysis of CYBA by BChE. These results are consistent with the inhibitory effects observed in plasma (Supplementary Figure S6). In subsequent selectivity assay, the catalytic effect of BChE on CYBA is not affected by various amino acids and ions (Figures 2G, H). These results clearly indicate that CYBA is a highly specific fluorescent substrate for BChE and CYBA is a promising molecular tool for detecting the BChE activity in complex bio-samples.

### 3.4 Metabolic characteristics of CYBA as a NIR fluorescence probe of BChE

**FIGURE 3:**
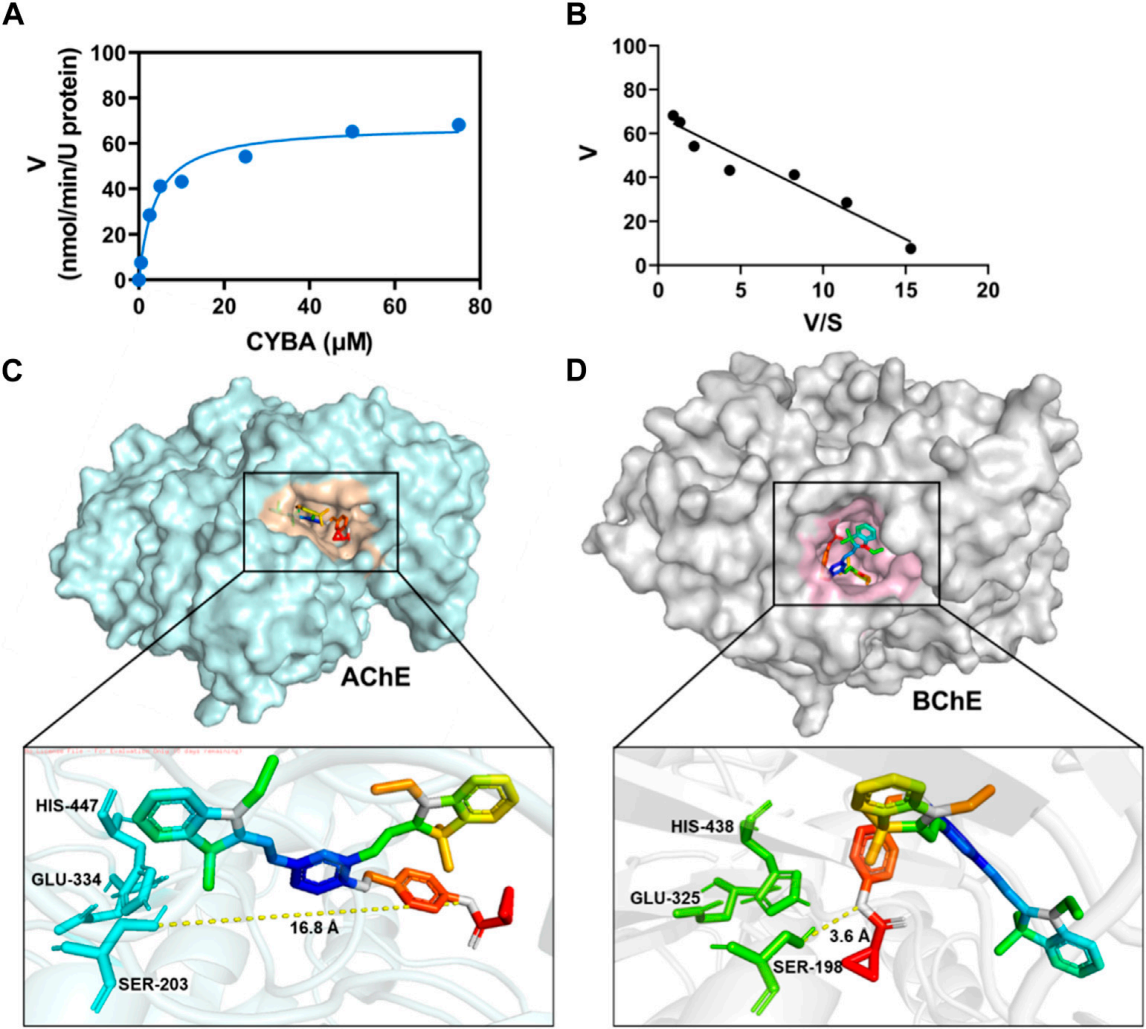
**(A)** Michaelis−Menten curve for **CYBA** (5 μM) catalysis by BChE (1 U/mL) for 30 min at 37 ^o^C in PBS buffer (pH 7.4) assessed using a fluorometric method. **(B)** Eadie-Hofstee plot (R^2^ = 0.9). The results are expressed as the mean ± SD (n = 3). λ_ex_/λ_em_ = 610/710 nm. Molecular docking of CYBA to AChE **(C)** or BChE **(D)**.

The metabolic behavior and binding location of CYBA in BChE can be effectively studied using a combination of enzymatic and computer docking techniques. Figures 3A, B demonstrated that the hydrolytic behavior of CYBA in BChE followed typical Michaelis-Menten kinetics, which is supported by the Eadie-Hofstee plot. CYBA showed high affinity and good responsiveness to BChE (*K*_*m*_ = 3.86 ± 0.86 µM; *V*_max_ = 68.28 ± 0.86 nmol/min/U BChE). The exceptional kinetic characteristics and properties of the probe formed the foundation for its subsequent utilization in the identification of intricate biological samples. Molecular docking simulation between the CYBA and AChE or BChE were further carried out (Figures 3C, D). It is obvious that CYBA fails to enter to the catalytic pocket of AChE, while CYBA could go into the catalytic pocket of BChE. The distance between CYBA and Ser198 is 3.6Å. The CYBA formed two hydrogen bonds with two amino acids (SER198, HIS438) in BChE and formed Pi-alkal with Trp82 and Trp231 (Supplementary Figure S7). These findings indicate that CYBA has the ability to form a stable complex with BChE and effectively conduct enzymatic cleavage.

### 3.5 Determination of BChE in human plasma

BChE is present in high quantities in plasma, with a concentration ranging from 2–5 mg/L (Pohanka, 2020; Jasiecki et al., 2021). Consequently, it is crucial to measure BChE activity in plasma in order to investigate the enzymatic metabolism of BChE utilizing plasma as a source of the enzyme. Within the plasma, the levels of BChE were significantly greater than those of AChE, CES, and pancreatic lipase. In fact, BChE levels were 235 times higher than CES1 levels (Supplementary Figure S8). In order to assess the effectiveness of the CYBA in properly measuring BChE levels in human plasma, we carried out spike-and-recovery experiments. As indicated in Table 1, the addition of standard BChE solutions at concentrations of 1, 10 and 20 U/L to the 100-fold diluted human plasma resulted in recovery rates ranging from 97.51% to 104.01% (Table 1). Similar results were found using the Ellman method. These acceptable recovery rates highlight the potential of CYBA in the precise measurement of BChE levels in human plasma.

**TABLE 1:** Recovery rates of BChE spiked into human plasma (n = 3).

Sample	BChE added (U/L)	Detected by CYBA (U/L)	Recovery (%)	RSD (%)	Detected by Ellman method (U/L)
Blank	0	22.10 ± 1.32	-	-	20.81 ± 2.21
1	1	23.05 ± 0.64	99.80 ± 2.76	2.76	22.01 ± 1.94
2	10	33.39 ± 1.68	104.01 ± 5.23	5.03	31.56 ± 1.67
3	20	41.05 ± 2.79	97.51 ± 6.62	6.79	41.89 ± 2.03

### 3.6 Bio-imaging applications in living cells

**FIGURE 4:**
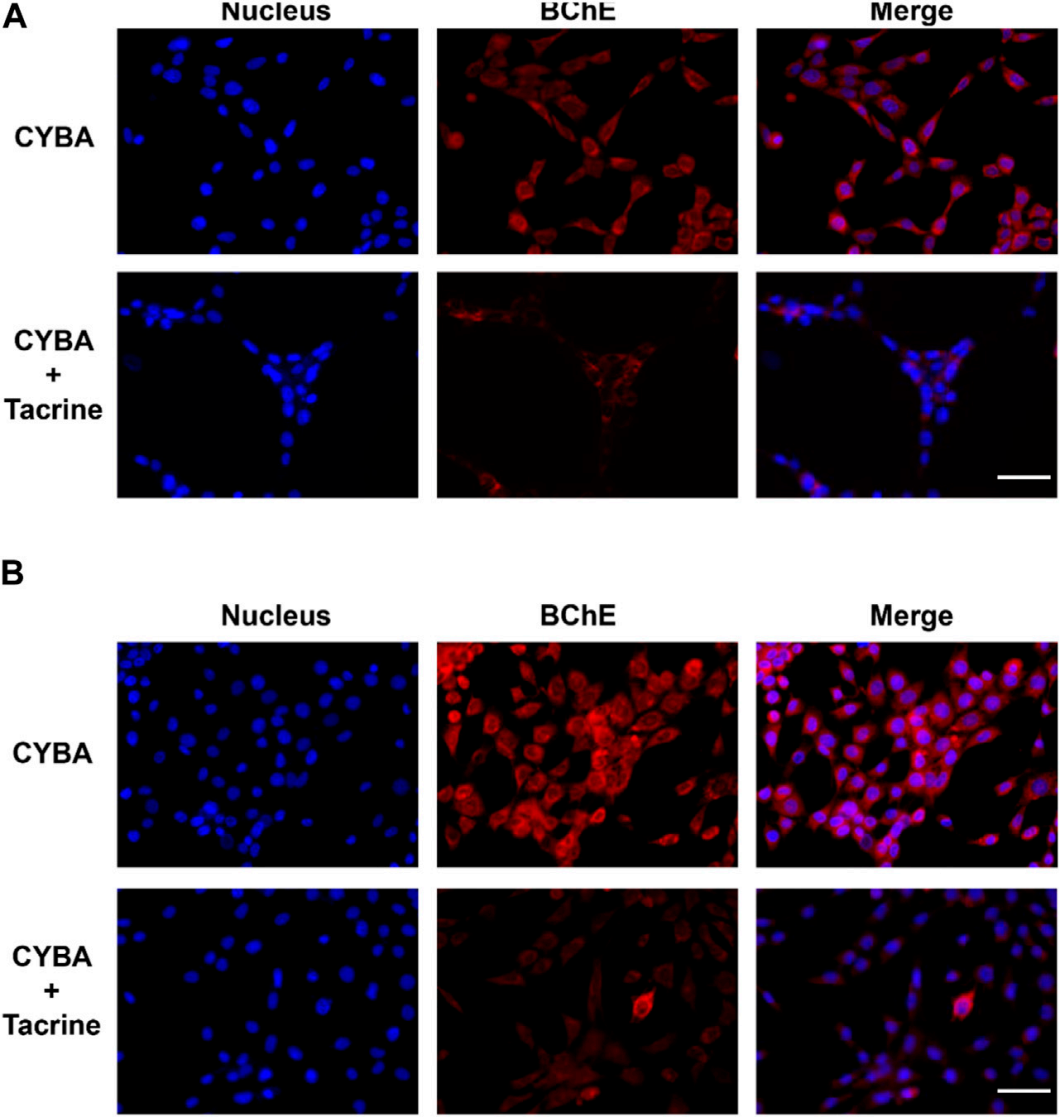
Fluorescence images of CYBA (10 μM) in living HT22 cells **(A)** and U87MG cells **(B)**. Control: cells incubated with 10 μM CYBA for 1 h and Hoechst 33342 (10 μM) for 20 min; TAC: cells were pre-treated with inhibitor tacrine (50 μM) for 1 h, then incubated with 10 μM CYBA for 1 h and Hoechst 33342 (10 μM) for 20 min. Blue channel for Hoechst 33342, 420–480 nm, red channel for CYBA, 660–730 nm. Scale bars = 20 μm.

Based on the above excellent performance, CYBA was then applied for visualizing cellular BChE activity. HT22 cells (mouse hippocampal neurons cells) and U87MG cells (human malignant glioblastoma cells) have been identified to exhibit BChE activity (https://www.proteinatlas.org/) (Salau et al., 2020). Therefore, these 2 cell lines were chosen as the model cell systems for CYBA fluorescence imaging experiments. HT22 cells and U87MG cells cultured with the probe CYBA showed bright intracellular red fluorescence (Figure 4). By contrast, the fluorescent signal was significantly reduced by adding tacrine (a specific inhibitor). Compared with the fluorescence intensity of HT22 cells, U87MG exhibited stronger fluorescence intensity. These results suggested that CYBA has a good cell-permeability and could also function in model cell systems efficiently.

### 3.7 High-throughput screening of BChE inhibitors

**FIGURE 5:**
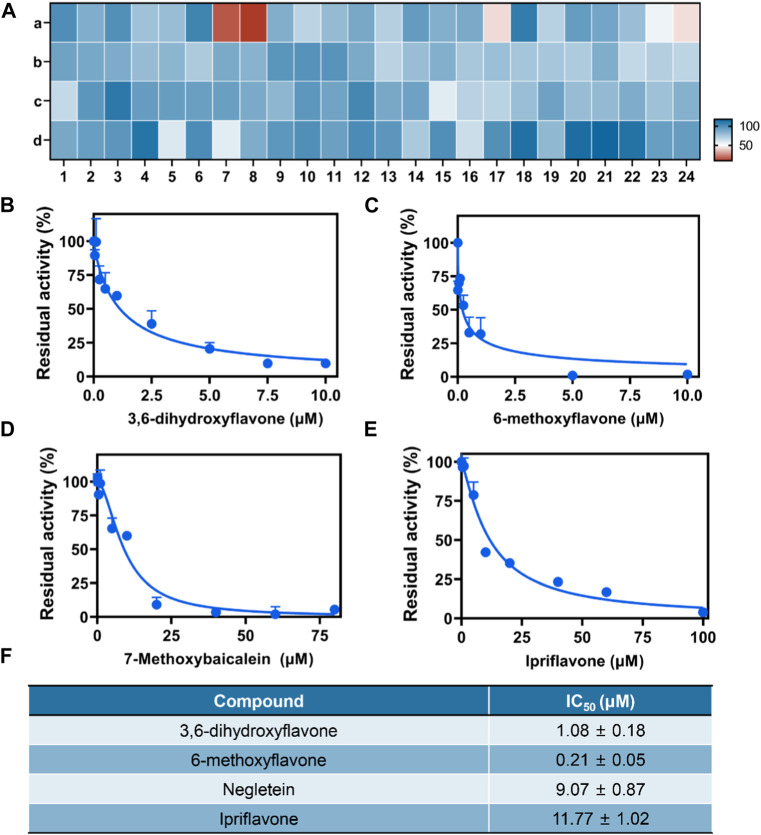
**(A)** Heatmap for high-throughput screening of 96 native compounds (10 μM) derived from herbs upon BChE inhibition, flavonoids **(A)**, anthraquinones **(B)**, ginsengs **(C)**, lignins **(D)**. Dose-inhibition curves of 3,6-dihydroxyflavone **(B)**, 6-methoxyflavone **(C)**, 7-methoxybaicalein **(D)**, Ipriflavone **(E)** against BChE-catelyzed BChE hydrolysis. **(F)** The IC_50_ value of four inhibitors. The results are expressed as the mean ± SD (n = 3). λ_ex_/λ_em_ = 610/710 nm.

We developed a high-throughput screening method for BChE inhibitors in order to identify potential candidates due to the superior sensitivity and anti-interference properties. A visual high-throughput assay was developed in this instance to screen for inhibitors of BChE. The assay utilized CYBA as a fluorogenic substrate and plasma as the enzyme source, with the screening conducted in a 96-well microplate. Afterwards, a comprehensive assessment was conducted on 96 compounds derived from four natural sources (flavonoids, anthraquinones, ginsengs, and lignans) to determine their ability to inhibit BChE (Figure 5A; Supplementary Table S1). The result clearly showed that four potent inhibitors display a notable inhibitory impact on BChE (Figure 5B–E; Supplementary Figure S8). As shown in Figures 5B–F, the IC_50_ values for 3,6-dihydroxyflavone, 6-methoxyflavone, 7-methoxybaicalein, Ipriflavone were 1.08 µM, 0.21 µM, 9.07 µM and 11.77 µM, respectively. In summary, the results indicated that the CYBA-based assay shows significant potential for visually screening BChE inhibitors in a high-throughput screening.

## 4 Conclusion

In summary, we designed and developed a novel NIR fluorescent probe (CYBA) based on a cyanine-skeleton for detecting BChE activity. CYBA showed a NIR fluorescence enhancement at 710 nm after being metabolized by BChE, and it possessed high selectivity and sensitivity toward BChE, where it was able to easily distinguish between AChE and BChE. Moreover, CYBA has been applied to trace BChE activity in living cells with good cell membrane permeability. Furthermore, CYBA was successfully used to visualize BChE activity in a high-throughput inhibitor screening. Results showed that flavonoids, inclunding 3,6-dihydroxyflavone, 6-methoxyflavone, 7-methoxybaicalein and ipriflavone, exhibited powerful inhibition on the activity of BChE in plasma. These results offer a novel, useful, and trustworthy approach for high-throughput screening of BChE inhibitors and quantitative determination of BChE activity in complicated biospecimens.

## Data Availability

The original contributions presented in the study are included in the article/Supplementary Material, further inquiries can be directed to the corresponding authors.
