# Supplementary material for: Rational design of a near-infrared fluorescent probe for monitoring butyrylcholinesterase activity and its application in development of inhibitors
Source: Front Bioeng Biotechnol. 2024 Apr 4;12:1387146. doi: 10.3389/fbioe.2024.1387146 (PMC11024273; doi:10.3389/fbioe.2024.1387146)
Supplement: Supplementary file 1 [file DataSheet1.docx]

***Supplementary Information***

**Rational design of a near-infrared fluorescent probe for monitoring butyrylcholinesterase activity and its application in development of inhibitors**

Hao Li^a,b†^, Xiao-Dong Li^a,†^, Chao-Hua Yan^a^, Zhen-Hua Ni^a^, Mu-Han Lü^b,^*, Li-Wei Zou^a,^*, Ling Yang^a,^*

^a^ Shanghai Frontiers Science Center of TCM Chemical Biology, Institute of Interdisciplinary Integrative Medicine Research, Shanghai University of Traditional Chinese Medicine, Shanghai 201203, China

^b^ Department of Gastroenterology, The Affiliated Hospital of Southwest Medical University, Luzhou 646000, China

†These authors contributed equally.

* Corresponding author. E-mail address: chemzlw@163.com (L.-W. Zou), yling@dicp.ac.cn (L. Yang), lvmuhan@swmu.edu.cn (M.-H. Lü)

**Table of Contents**

1. **Synthesis and structural characterization………………………..……..…….........………….3**
2. **Figures……………………………………………………………..….……..……….………….7**

2.1 Figure S1 The absorption spectra and fluorescence spectra of TBBO and TBO**………....……7**

2.2 Figure S2 The stability of CUBA, CYBA, TBBO and TBO…………………………….…..7

2.3 Figure S3 The emission spectra of TBO in various solvents……………………………..….8

2.4 Figure S4 Effects of pH values on the fluorescence intensity of CYBA and TBO……….….8

2.5 Figure S5 Representative LC-UV chromatograms of CYBA and TBO……………….……..8

2.6 Figure S6 Chemical inhibition of different inhibitors………………………………..….……9

2.7 Figure S7 Docking molecular interaction between CYBA and BChE.………………………9

2.8 Figure S8 The concentrations of several common hydrolytic enzymes in plasma………..…10

2.9 Figure S9 Cell toxicity of CYBA in HT22 cells…………………………………….…...…10

2.10 Figure S10 Cell toxicity of CYBA in U87MG cells……………………………….…...…10

2.11 Chemical Structural Formulas of four Potent Inhibitors………………...……….………..11

1. **Tables…………………………………………………………………………….….………….11**

3.1 Table S1 Properties of representive fluorescent probes of BChE…………….………….…11

3.2 Table S1 Residual activity of 96 natural products…………………………….……………12

1. **NMR spectra and HRMS spectrum for NIR fluorescent probes……………….…….……15**
2. **HRMS spectrum of compounds………………………………………………….…..………20**
3. **Synthesis of Compounds**

**
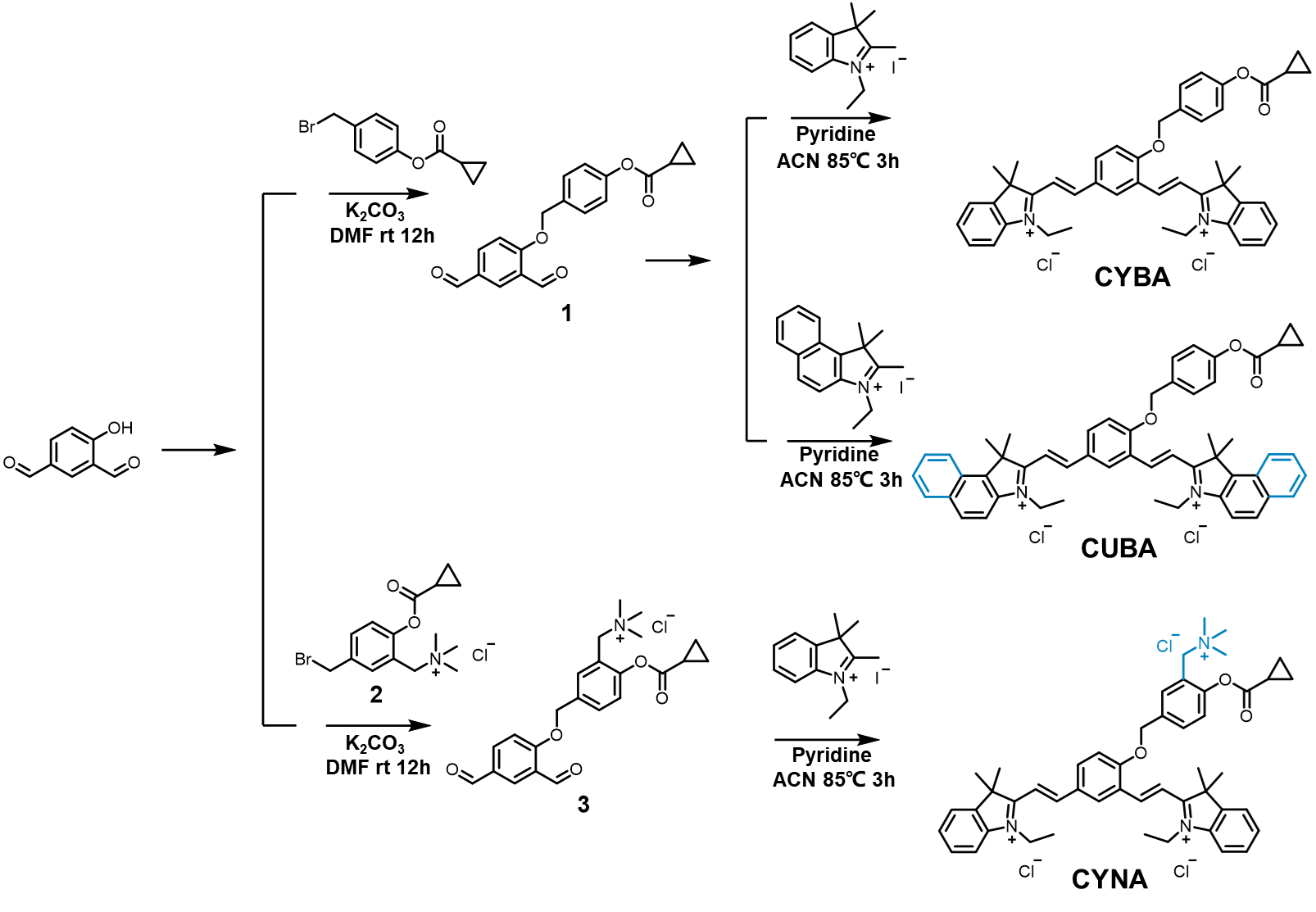
**

**Synthesis of CYBA**

4-hydroxyisophthalaldehyde (150.5 mg, 1.0 mmol) and K_2_CO_3_ (208.2 mg, 1.5 mmol) were dissolved in 5 mL of dry DMF. The mixture was then stirred at room temperature while being protected with argon, then 4-(bromomethyl)phenyl cyclopropane carboxylate (85.5 mg, 0.40 mmol) was added and the reaction mixture was stirred under argon overnight. After the reaction was completed, the reaction mixture was diluted with 100 mL of ethyl acetate (EtOAc) and washed successively with two 100-mL portions of brine and 50 mL of water. The organic layer was dried (anhydrous MgSO_4_) and concentrated under diminished pressure. The solvent was removed under vacuum, and the product was purified by silica gel column chromatography (petroleum ether/ethyl acetate = 10:1-4:1). Compound **1** was obtained as 198.8 mg of white oil with a yield of 61.3%.

1-Ethyl-2,3,3-trimethyl-3H-indolium (378.2 mg, 1.2 mmol) and pyridine (103.1 mg, 1.2 mmol) were dissolved in 10 mL of acetonitrile. The mixture was gradually heated to 85°C over 10 minutes. Afterward, compound 1 (324.3 mg, 1.0 mmol) was added and the reaction mixture was stirred at 85°C for 6 h. The resulting mixture was then diluted with 20 mL of water and extracted three times with 30 mL of ethyl acetate. The organic layer was dried over anhydrous MgSO_4_. The solvent was evaporated under vacuum, and the product was purified by silica gel column chromatography using a mixture of dichloromethane and methanol (30:1-20:1) as the eluent, **CYBA** was obtained as 186.9 mg of dark -black crystals with a yield of 25.4%. ^1^H NMR (600 MHz, DMSO-d6) δ 9.02 (d, J = 2.3 Hz, 1H), 8.65 (dd, J = 8.9, 2.2 Hz, 1H), 8.53 (dd, J = 21.5, 16.3 Hz, 2H), 7.99-7.97 (m, 2H), 7.95-7.93 (m, 1H), 7.91 (dd, J = 5.7, 3.1 Hz, 1H), 7.87 (d, J = 16.5 Hz, 1H), 7.78 (d, J = 16.3 Hz, 1H), 7.70 (d, J = 8.5 Hz, 2H), 7.66 (dd, J = 5.9, 2.9 Hz, 5H), 7.36 – 7.19 (m, 2H), 5.76 (s, 2H), 5.52 (s, 2H), 4.81 (d, J = 7.3 Hz, 2H), 4.52 (d, J = 7.4 Hz, 2H), 1.88 (s, 7H), 1.78 (s, 7H), 1.51 (t, J = 7.2 Hz, 4H), 1.34 (t, J = 7.3 Hz, 4H), 1.14–1.08 (m, 4H), 1.07–1.02 (m, 3H).^13^C NMR (151 MHz, DMSO) δ 182.00, 181.63, 173.39, 173.37, 163.25, 162.69, 152.74, 151.15, 148.25, 144.41, 144.32, 140.93, 140.88, 136.82, 135.92, 133.65, 130.27, 130.25, 129.46, 128.32, 123.81, 123.64, 122.67, 122.63, 122.50, 115.86, 115.67, 115.04, 114.98, 112.19, 52.79, 52.76, 27.31, 26.22, 26.20, 14.28, 13.87, 13.10, 9.62. HRMS (ESI) Calcd. for C_45_H_48_N_2_O_3_^2+^ ([M]^+^) 332.1827, Found 332.1826.

**Synthesis of CUBA**

1,1,2,3-tetramethyl-1H-benzo[e]indol-3-ium iodide (438.5 mg, 1.2 mmol) and pyridine (103.1 mg, 1.2 mmol) were dissolved in 10 mL of acetonitrile. The resulting mixture was then slowly heated over a period of 10 minutes until it reached a temperature of 85℃. Once the desired temperature was reached, compound **1** (324.3 mg, 1.0 mmol) was carefully added, and the reaction mixture was stirred at 85℃ for 6 hours. After completion of the reaction, the resulting mixture was diluted with 20 mL of water and subjected to three extractions using 30 mL portions of ethyl acetate each time. The combined organic layers were then dried using anhydrous MgSO_4_ as a drying agent. The solvent was subsequently removed under vacuum, and the obtained product was purified using silica gel column chromatography employing a dichloromethane/methanol gradient (ranging from 50:1 to 20:1). **CUBA** was obtained as 147.1 mg of dark -black crystals with a yield of 17.6%. ^1^H NMR (600 MHz, DMSO-d6) δ 9.01 (d, J = 2.2 Hz, 1H), 8.69–8.63 (m, 2H), 8.62–8.57 (m, 2H), 8.47 (t, J = 7.8 Hz, 2H), 8.34 (dd, J = 9.0, 5.0 Hz, 2H), 8.26 (d, J = 8.3 Hz, 2H), 8.17 (dd, J = 10.8, 8.9 Hz, 2H), 7.93 (d, J = 16.5 Hz, 1H), 7.87–7.82 (m, 3H), 7.77 (td, J = 7.5, 3.6 Hz, 2H), 7.74–7.71 (m, 2H), 7.68 (d, J = 9.0 Hz, 1H), 7.35–7.28 (m, 2H), 5.54 (s, 2H), 4.94 (q, J = 7.1 Hz, 2H), 4.68 (q, J = 7.3 Hz, 2H), 2.10 (s, 6H), 1.99 (s, 6H), 1.58 (t, J = 7.3 Hz, 3H), 1.44 (t, J = 7.3 Hz, 3H), 1.12 (d, J = 9.4 Hz, 4H), 1.07 (dd, J = 4.6, 2.8 Hz, 2H). ^13^C NMR (151 MHz, DMSO) δ 182.64, 182.24, 173.48, 162.43, 151.63, 151.16, 146.84, 139.31, 139.18, 138.63, 136.69, 135.18, 133.81, 133.74, 131.72, 130.55, 130.20, 128.98, 128.45, 127.98, 127.82, 127.25, 127.21, 124.02, 123.76, 123.67, 122.76, 115.03, 114.46, 113.76, 111.80, 71.30, 54.38, 54.36, 49.21, 43.52, 43.11, 27.31, 26.09, 26.03, 14.52, 14.18, 13.13, 9.65. HRMS (ESI) Calcd. for C_53_H_52_N_2_O_3_^2+^ ([M]^+^) 382.1983, Found 382.1988.

**Synthesis of CYNA**

To initiate the reaction, 4-hydroxyisophthalaldehyde (150.5 mg, 1.0 mmol) and K_2_CO_3_ (208.2 mg, 1.5 mmol) were dissolved in 5 mL of dry DMF. The resulting mixture was stirred at room temperature under an argon atmosphere. Subsequently, compound **2** (362.5 mg, 1.0 mmol) was added, and the reaction mixture was stirred overnight, still under an argon atmosphere. Once the reaction was completed, the reaction mixture was diluted with 100 mL of EtOAc and sequentially washed with two portions of 80 mL brine and 50 mL water. The organic layer was then dried using anhydrous MgSO_4_ and concentrated under reduced pressure. The solvent was removed under vacuum, and the resulting product was purified by silica gel column chromatography using a petroleum ether/ethyl acetate gradient (ranging from 4:1 to 1:1).

Compound **3** was obtained as 354.1 mg of white oil with a yield of 82.1%. 1-Ethyl-2,3,3-trimethyl-3H-indolium (378.2 mg, 1.2 mmol) and pyridine (103.1 mg, 1.2 mmol) were dissolved in 15 mL of acetonitrile. The mixture was gradually heated to 85°C over 10 minutes. Afterward, compound 3 (431.9 mg, 1.0 mmol) was added and the reaction mixture was stirred at 85°C for 6 h. After completion of the reaction, the resulting mixture was diluted with 20 mL of water and subjected to three extractions using 30 mL ethyl acetate. The organic layer was then dried using anhydrous MgSO_4_. The solvent was evaporated under vacuum, and the obtained product was purified by silica gel column chromatography using a gradient eluent mixture of dichloromethane and methanol (ranging from 60:1 to 20:1). Through this purification process, **CYNA** was obtained as 185.5 mg of purple-black crystals with a yield of 22.3%. ^1^H NMR (600 MHz, DMSO-d6) δ 8.41 (d, J = 16.2 Hz, 1H), 8.16 (d, J = 2.2 Hz, 1H), 8.12 (dd, J = 8.6, 2.2 Hz, 1H), 7.89 (ddd, J = 16.0, 7.0, 1.4 Hz, 2H), 7.67 – 7.57 (m, 2H), 7.54 (d, J = 16.1 Hz, 1H), 7.19 – 7.05 (m, 3H), 6.93 (d, J = 8.5 Hz, 1H), 6.79 (t, J = 7.4 Hz, 1H), 6.62 (d, J = 7.9 Hz, 1H), 5.99 (d, J = 10.3 Hz, 1H), 4.69 (q, J = 7.2 Hz, 2H), 3.31 (d, J = 7.4 Hz, 1H), 3.19 (dq, J = 13.9, 6.8 Hz, 1H), 1.78 (d, J = 8.3 Hz, 9H), 1.45 (t, J = 7.2 Hz, 3H), 1.24 (s, 3H), 1.16–1.04 (m, 6H), 0.85 (qd, J = 7.5, 4.4 Hz, 1H). ^13^C NMR (151 MHz, DMSO) δ 181.01, 158.82, 154.00, 153.58, 146.29, 143.69, 140.46, 135.79, 133.40, 130.34, 129.08, 129.02, 128.46, 127.59, 127.13, 123.07, 121.71, 121.25, 119.18, 118.72, 115.96, 114.74, 109.64, 106.35, 106.04, 52.18, 51.95, 25.97, 25.89, 25.76, 19.51, 13.88, 13.62, 13.43. HRMS (ESI) Calcd. for C_49_H_58_N_3_O_3_^3+^ ([M]^+^) 245.4821, Found 245.4822.


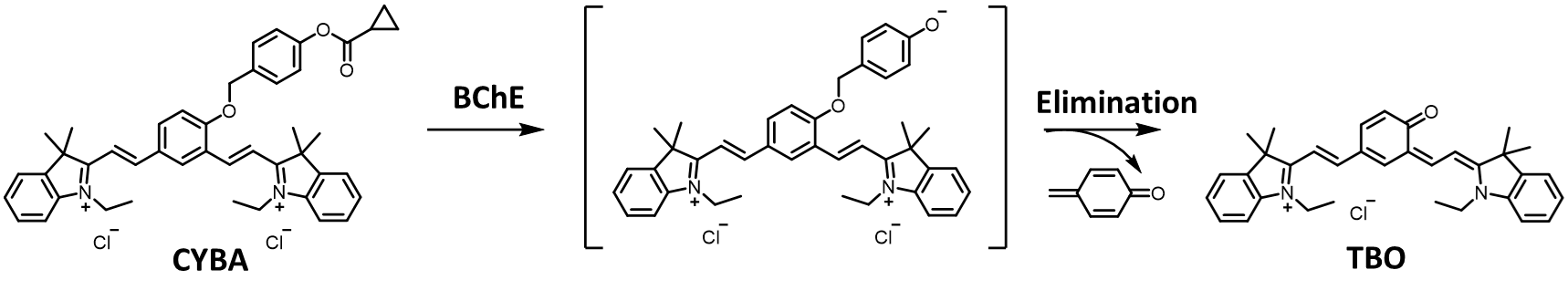


**TBO**

^1^H NMR (600 MHz, DMSO-d6) δ 8.41 (d, J = 16.1 Hz, 1H), 8.17 (s, 1H), 8.12 (dd, J = 8.6, 2.2 Hz, 1H), 7.94–7.80 (m, 2H), 7.71–7.59 (m, 2H), 7.53 (d, J = 16.1 Hz, 1H), 7.20–7.04 (m, 3H), 6.93 (d, J = 8.5 Hz, 1H), 6.78 (t, J = 7.4 Hz, 1H), 6.62 (d, J = 7.9 Hz, 1H), 5.99 (d, J = 10.3 Hz, 1H), 5.76 (s, 2H), 4.69 (q, J = 7.2 Hz, 2H), 3.34–3.28 (m, 1H), 3.19 (dq, J = 14.1, 6.8 Hz, 1H), 2.51 (q, J = 1.9 Hz, 3H), 1.79 (s, 7H), 1.45 (t, J = 7.2 Hz, 3H), 1.24 (s, 4H), 1.15–1.00 (m, 6H). ^13^C NMR (151 MHz, DMSO) δ 181.49, 159.31, 154.45, 146.77, 144.17, 140.94, 136.27, 133.93, 130.76, 129.56, 129.51, 128.93, 128.06, 127.59, 123.56, 122.19, 121.73, 119.68, 119.20, 116.43, 115.23, 110.10, 106.83, 106.52, 55.40, 52.65, 52.43, 44.24, 42.28, 26.45, 26.25, 22.69, 22.07, 21.54, 19.99, 14.36, 14.12. HRMS (ESI) Calcd. for C_42_H_41_N_2_O^+^ ([M]^+^) 589.3213, Found 589.3223.


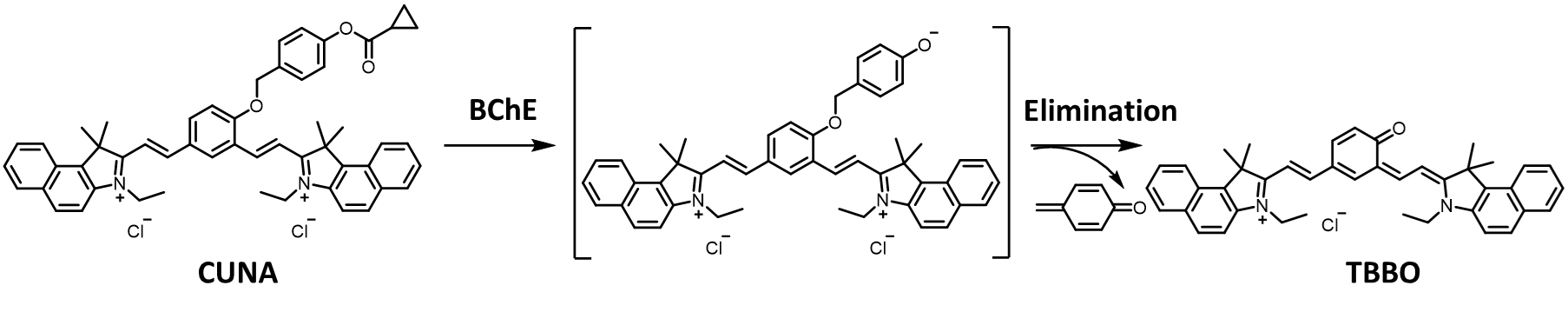


**TBBO**

^1^H NMR (600 MHz, DMSO-d6) δ 8.50 (d, J = 16.2 Hz, 1H), 8.43 (d, J = 8.5 Hz, 1H), 8.30 (d, J = 8.9 Hz, 1H), 8.22 (d, J = 8.2 Hz, 1H), 8.18 (d, J = 5.0 Hz, 1H), 8.12 (ddd, J = 13.5, 8.8, 1.8 Hz, 2H), 7.92 (d, J = 8.6 Hz, 1H), 7.88–7.86 (m, 1H), 7.83–7.80 (m, 2H), 7.73 (ddd, J = 8.1, 6.8, 1.1 Hz, 1H), 7.56 (dd, J = 16.3, 1.5 Hz, 1H), 7.43 (ddd, J = 8.3, 6.7, 1.4 Hz, 1H), 7.28–7.19 (m, 2H), 7.15 (d, J = 8.6 Hz, 1H), 6.90 (d, J = 8.5 Hz, 1H), 6.08 (d, J = 10.3 Hz, 1H), 4.87–4.61 (m, 2H), 3.46–3.41 (m, 1H), 2.03 (d, J = 7.6 Hz, 7H), 1.58 (s, 3H), 1.51 (t, J = 7.2 Hz, 3H), 1.29 (s, 3H), 1.17 (t, J = 7.1 Hz, 3H).^13^C NMR (101 MHz, DMSO) δ 182.12, 159.31, 153.24, 144.89, 138.66, 138.60, 133.83, 133.55, 131.53, 130.76, 130.51, 130.12, 129.86, 129.47, 129.44, 129.16, 128.89, 127.61, 127.54, 127.29, 127.01, 124.83, 123.56, 121.90, 121.39, 121.17, 119.51, 116.29, 113.52, 110.56, 109.64, 107.66, 54.19, 54.05, 37.80, 34.66, 34.40, 27.02, 26.10, 25.25, 24.49, 21.44, 21.07, 14.85, 14.34. HRMS (ESI) Calcd. for C_34_H_37_N_2_O^+^ ([M]^+^) 489.2900, Found 489.2905.

1. **Figures**

**
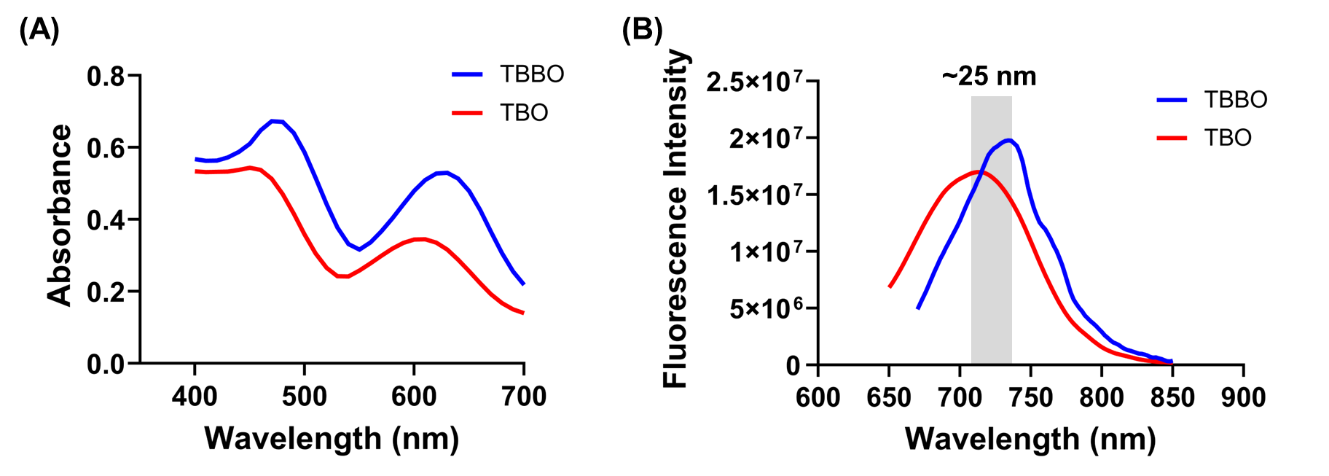
**

**Figure S1** The absorption spectra (A) and fluorescence spectra (B) of TBBO (20 μM) and TBO (20 μM) in PBS–ethanol (v/v =1:1, pH 7.4).


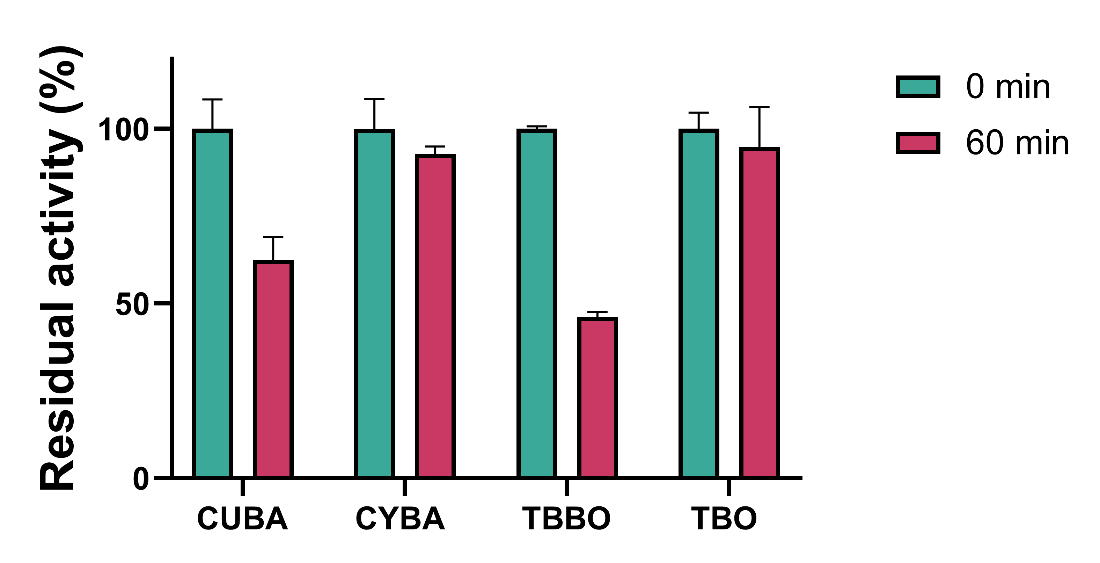


**Figure S2** The stability of CUBA (20 μM), CYBA (20 μM), TBBO (20 μM) and TBO (20 μM) at 37 ℃ in 0 min and 60 min.

**
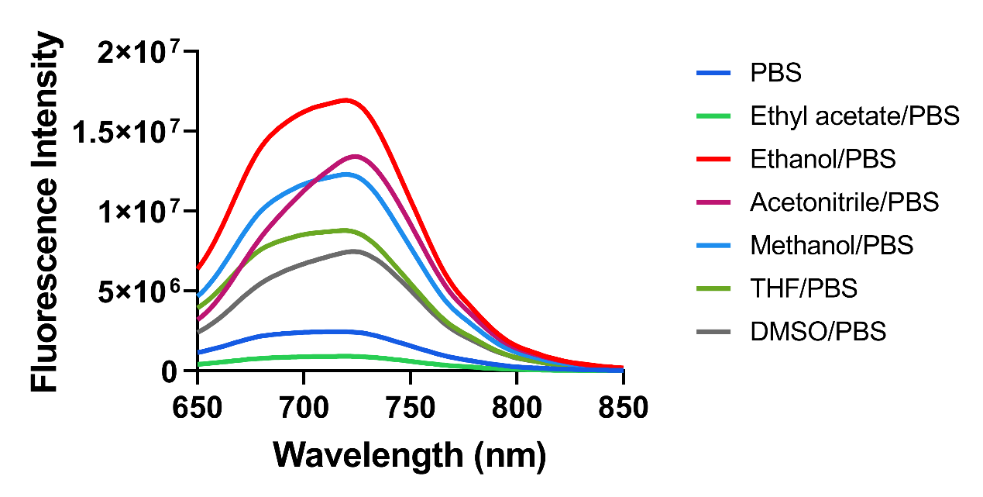
**

**Figure S3** The emission spectra of TBO (20 μM) in various solvents.

**
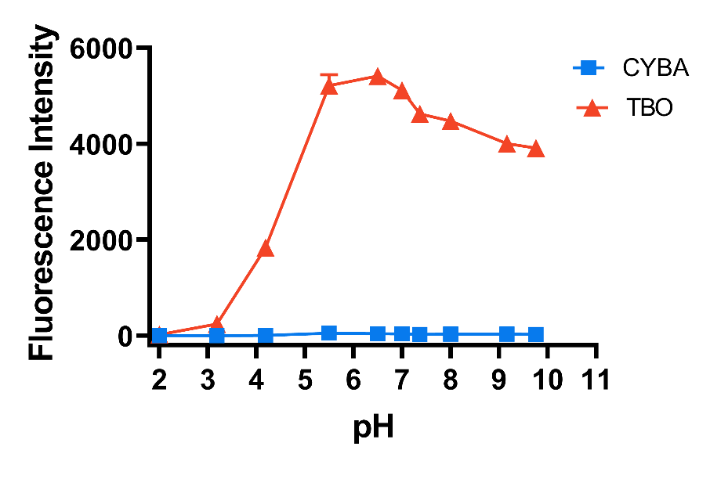
**

**Figure S4** Effects of pH values on the fluorescence intensity of CYBA (20 μM) and TBO (20 μM). The measurements were performed in Tris-HCl buffer with different pH values adjusted by NaOH.

**
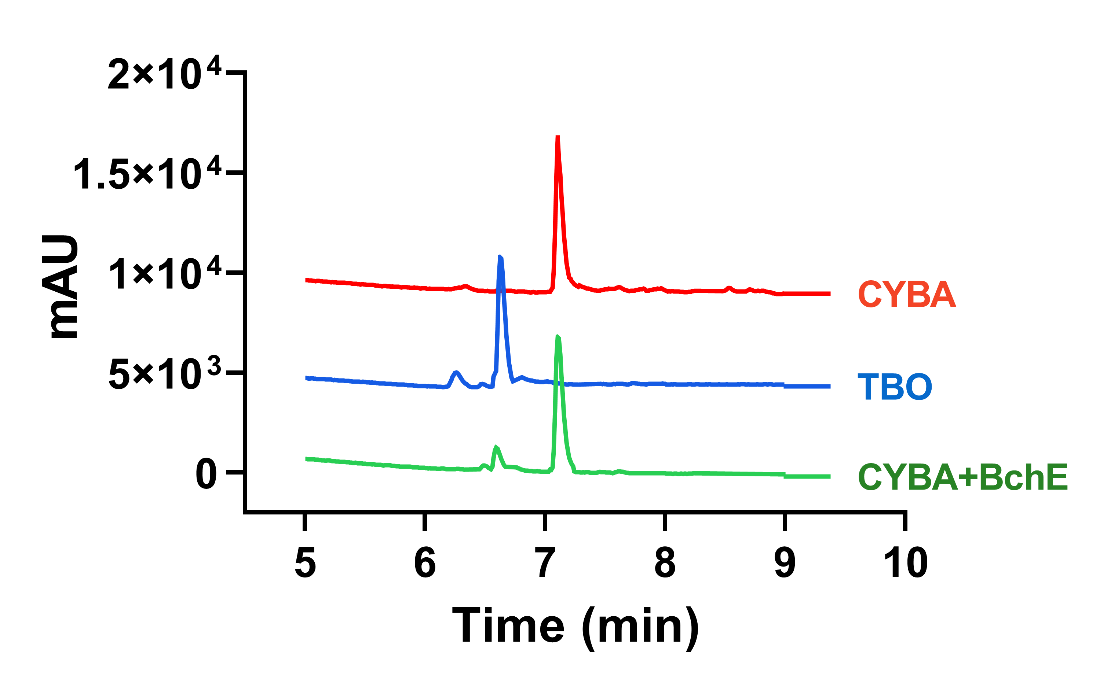
**

**Figure S5** Representative LC-UV chromatograms of CYBA (20 μM), TBO (20 μM), and CYBA (20 μM) incubation with BChE at 37 °C for 30 min, UV detector wavelength was set at 254 nm.


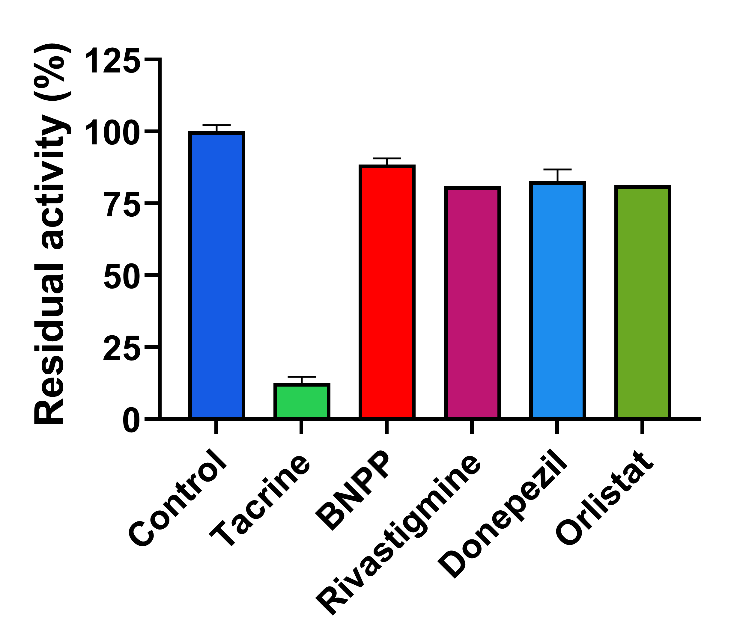


**Figure S6** Chemical inhibition of different inhibitors (10 μM) towards CYBA (5 μM) metabolism.

**
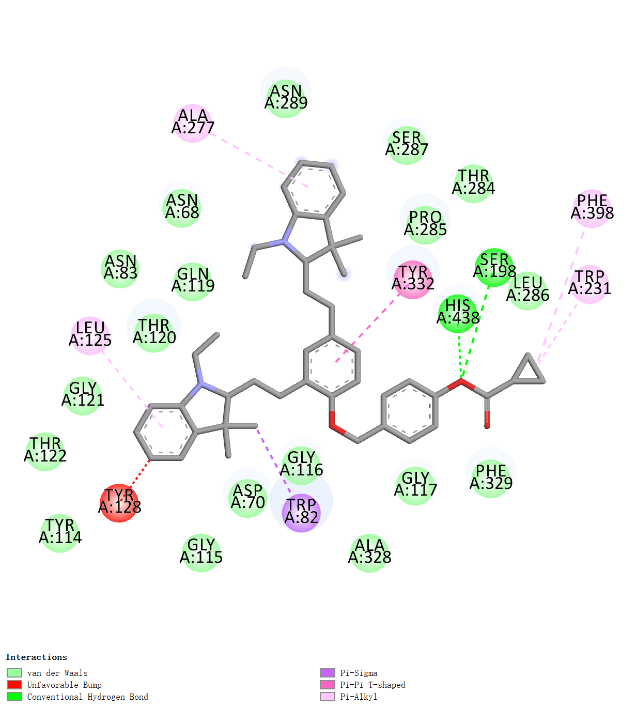
**

**Figure S7** Docking molecular interaction between CYBA and BChE.


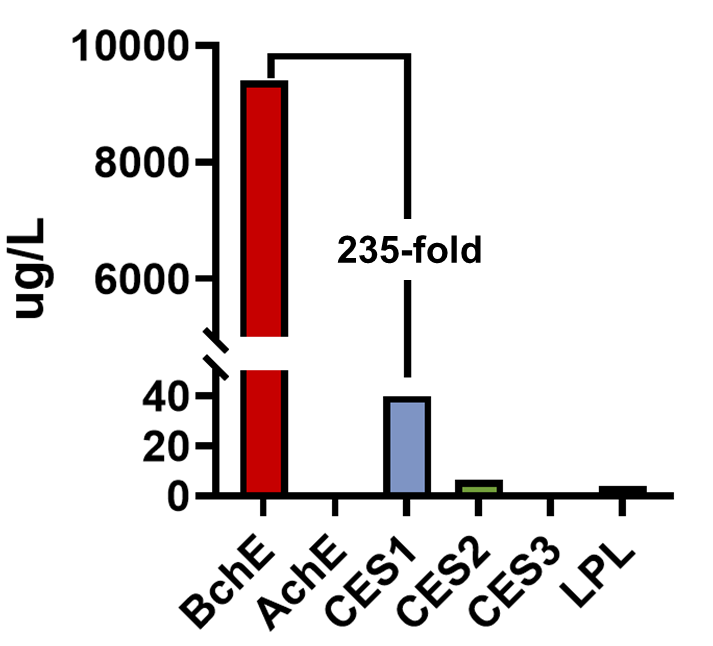


**Figure S8** The concentrations of several common hydrolytic enzymes in plasma.

**
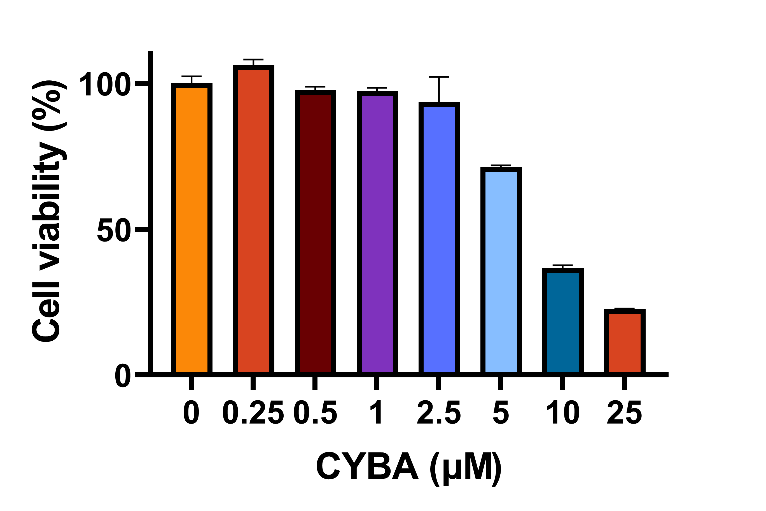
**

**Figure S9** Cell toxicity of CYBA in HT22 cells.

**
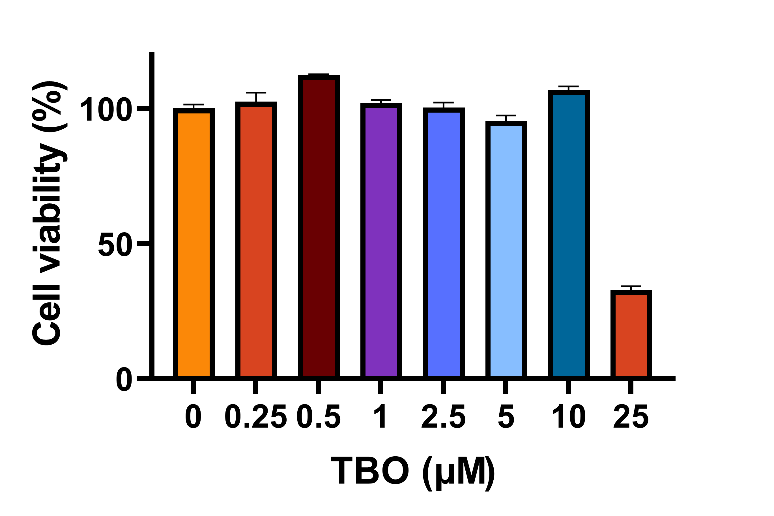
**

**Figure S10** Cell toxicity of CYBA in U87MG cells.

**Figure S11** Chemical structural formulas of four potent inhibitors.

**3. Tables**

**3.1 Table S1** Properties of representive fluorescent probes of BChE

| **Probe** | **Sensing mode** | **λex/λem (nm)** | **Stokes shifts (nm)** | **LOD** | **Reference** |
| --- | --- | --- | --- | --- | --- |
|  | off-on | 670/708 | 38 | 4.35 U/L | [1] |
|  | off-on | 458/528 | 70 | 0.075 μg/mL | [2] |
|  | on-off | - | - | - | [3] |
|  | ratiometric | 637/816(allfor λem) | 179 (blue shift) | 0.24 ug/mL | [4] |
|  | off-on | 610/710 | 100 | 0.009 U/mL | This work |

[1] Ma J, Lu X, Zhai H, et al. Rational design of a near-infrared fluorescence probe for highly selective sensing butyrylcholinesterase (BChE) and its bioimaging applications in living cell. Talanta. 2020, 1;219:121278.

[2] Zhang WD, Zhang JM, et al. A far-red/near-infrared fluorescence probe with large Stokes shift for monitoring butyrylcholinesterase (BChE) in living cells and in vivo. Anal Chim Acta. 2022, 1;1235:340540.

[3] Yang SH, Sun Q, Xiong H, et al. Discovery of a butyrylcholinesterase-specific probe via a structure-based design strategy. Chem Commun (Camb). 2017, 4;53(28):3952-3955.

[4] Yuan W, Wan C, Zhang J, et al. Near-infrared ratiometric fluorescent strategy for butyrylcholinesterase activity and its application in the detection of pesticide residue in food samples and biological imaging. Spectrochim Acta A Mol Biomol Spectrosc. 2023, 5;297:122719.

**3.2 Table S2** Residual activity of 96 natural products derived from herbs (10 μM, final concentration) against BChE-catalyzed CYBA hydrolysis.

| No. | Compound | CAS | MW | Relative activity (%) |
| --- | --- | --- | --- | --- |
| Flavonoids | | | | |
| a1 | Hispidulin | 1447-88-7 | 300.26 | 68.99 |
| a2 | 3',4'-dihydroxyflavone | 4143-64-0 | 254.24 | 79.39 |
| a3 | Artemisinin | 491-54-3 | 300.26 | 61.58 |
| a4 | Isovitexin | 29702-25-8 | 432.38 | 63.41 |
| a5 | 7,8-dihydroxyflavone | 38183-03-8 | 254.24 | 83.78 |
| a6 | 3,6-dihydroxyflavone | 108238-41-1 | 254.24 | 12.75 |
| a7 | 6-methoxyflavone | 26964-24-9 | 252.26 | 8.22 |
| a8 | Wogonin | 632-85-9 | 284.26 | 68.57 |
| a9 | 7,4'-dihydroxyflavone | 2196-14-7 | 254.24 | 55.54 |
| a10 | Alpinetin | 36052-37-6 | 270.28 | 64.24 |
| a11 | Isoxanthohumol | 70872-29-6 | 354.4 | 70.77 |
| a12 | Neoliquiritin | 5088-75-5 | 418.39 | 56.88 |
| a13 | Nobiletin | 478-01-3 | 402.39 | 75.39 |
| a14 | 4',5-dihydroxy-7-methoxyflavone | 437-64-9 | 284.26 | 68.43 |
| a15 | Acacetin | 480-44-4 | 284.26 | 62.90 |
| a16 | Glycitein | 40957-83-3 | 284.26 | 70.09 |
| a17 | Negletein | 29550-13-8 | 284.26 | 33.92 |
| a18 | Diosmetin | 520-34-3 | 300.26 | 86.59 |
| a19 | Demethyl tetrandrine | 33889-68-8 | 608.72 | 62.96 |
| a20 | D-tetrandrine | 518-34-3 | 622.75 | 64.88 |
| a21 | Myricitrin | 17912-87-7 | 464.38 | 71.83 |
| a22 | Ipriflavone | 35212-22-7 | 280.32 | 44.03 |
| a23 | Herbacetin | 527-95-7 | 302.24 | 57.16 |
| a24 | Taxifolin | 480-18-2 | 304.25 | 53.68 |
| Anthraquinones | | | | |
| b1 | 1,4-dihydroxyanthraquinone | 81-64-1 | 240.21 | 91.32 |
| b2 | Chrysophanic acid | 481-74-3 | 254.24 | 88.62 |
| b3 | Sennoside B | 128-57-4 | 862.74 | 87.20 |
| b4 | 2,6-dihydroxyanthraquinone | 84-60-6 | 240.21 | 80.03 |
| b5 | Sennoside A | 81-27-6 | 862.74 | 82.76 |
| b6 | Vat blue 4 | 81-77-6 | 442.42 | 73.26 |
| b7 | 1,4-naphthoquinone | 130-15-4 | 158.15 | 87.78 |
| b8 | 1,3-dihydroxyanthraquinone | 518-83-2 | 240.21 | 83.88 |
| b9 | 1,8-diaminoanthraquinone | 129-42-0 | 238.24 | 97.21 |
| b10 | 1,8-dihydroxyanthraquinone | 117-10-2 | 240.21 | 98.29 |
| b11 | 1-Hydroxy anthraquinone | 129-43-1 | 224.21 | 97.83 |
| b12 | 2-bromoanthraquinone | 572-83-8 | 287.11 | 87.78 |
| b13 | 2-Methyl anthraquinone | 84-54-8 | 222.24 | 69.64 |
| b14 | Anthraquinone | 84-65-1 | 208.21 | 79.05 |
| b15 | Emodin-3-methyl ether | 521-61-9 | 284.26 | 85.61 |
| b16 | 1,5-dihydroxyanthraquinone | 117-12-4 | 240.21 | 74.83 |
| b17 | 2-Ethyl anthraquinone | 84-51-5 | 236.27 | 78.44 |
| b18 | Solvent violet 13 | 81-48-1 | 329.35 | 75.62 |
| b19 | Anthraquinone-2-carboxylic acid | 117-78-2 | 252.22 | 75.03 |
| b20 | 1,5-diamino-4,8-dihydroxyanthraquinone | 145-49-3 | 270.24 | 73.16 |
| b21 | Alizarin complexone | 3952-78-1 | 385.32 | 86.48 |
| b22 | Hypericin | 548-04-9 | 504.45 | 64.52 |
| b23 | Aloe emodin | 481-72-1 | 270.24 | 65.78 |
| b24 | 2-chloroanthraquinone | 131-09-9 | 242.66 | 57.30 |
| Ginsengs | | | | |
| c1 | Ginsenoside Rb3 | 68406-26-8 | 1079.27 | 66.68 |
| c2 | Ginsenoside Rd | 52705-93-8 | 947.15 | 96.49 |
| c3 | 20R-Ginsenoside Rh2 | 112246-15-8 | 622.88 | 109.98 |
| c4 | Ginsenoside Rg1 | 22427-39-0 | 801.01 | 93.94 |
| c5 | Ginsenoside Rb2 | 11021-13-9 | 1079.27 | 93.64 |
| c6 | Ginsenoside Rg2 | 52286-74-5 | 785.03 | 92.56 |
| c7 | Notoginsenoside R1 | 80418-24-2 | 933.14 | 92.72 |
| c8 | Ginsenoside Rh4 | 174721-08-5 | 620.86 | 87.80 |
| c9 | Ginsenoside F2 | 62025-49-4 | 785.01 | 82.27 |
| c10 | Compound k | 39262-14-1 | 622.87 | 95.96 |
| c11 | Dammarenediol | 14351-29-2 | 444.73 | 90.79 |
| c12 | Ginsenoside F3 | 62025-50-7 | 770.99 | 103.08 |
| c13 | Protopanaxatriol | 32773-56-1 | 476.7 | 88.44 |
| c14 | Ginsenoside Rf | 52286-58-5 | 801.02 | 91.46 |
| c15 | Ginsenoside F1 | 53963-43-2 | 638.87 | 58.49 |
| c16 | Ginsenoside F4 | 181225-33-2 | 767.01 | 70.07 |
| c17 | Panaxatriol | 32791-84-7 | 476.73 | 69.55 |
| c18 | Ginsenoside Rb1 | 41753-43-9 | 1109.29 | 78.71 |
| c19 | Ginsenoside Rc | 11020-14-0 | 1079.27 | 91.45 |
| c20 | Ginsenoside Ro | 34367-04-9 | 957.11 | 79.60 |
| c21 | (20S)-Protopanaxatriol | 34080-08-5 | 476.74 | 80.48 |
| c22 | Panaxadiol | 19666-76-3 | 460.73 | 86.94 |
| c23 | Ginsenoside Rh3 | 105558-26-7 | 604.86 | 77.57 |
| c24 | Ginsenoside Rh2 | 78214-33-2 | 622.88 | 84.72 |
| Lignins | | | | |
| d1 | Schisandrin A | 61281-38-7 | 416.51 | 88.44 |
| d2 | Schisandrin | 7432-28-2 | 432.51 | 93.06 |
| d3 | Bifendate | 73536-69-3 | 418.35 | 96.47 |
| d4 | 4'-demethylpodophyllotoxin | 40505-27-9 | 400.38 | 111.75 |
| d5 | Tetrahydrocurcumin | 36062-04-1 | 372.41 | 60.01 |
| d6 | Podophyllotoxin | 518-28-5 | 414.41 | 100.81 |
| d7 | Bis(4-hydroxycinnamoyl)methane | 33171-05-0 | 308.33 | 58.29 |
| d8 | Schisandrin C | 61301-33-5 | 384.42 | 86.84 |
| d9 | Etoposide | 33419-42-0 | 588.56 | 95.94 |
| d10 | Teniposide | 29767-20-2 | 656.65 | 99.54 |
| d11 | 2-(1,3-Benzodioxol-5-yloxy)ethano | 109962-82-5 | 182.17 | 92.00 |
| d12 | 5-(Dodecyloxy)-1,3-benzodioxole | 87590-48-5 | 306.44 | 101.34 |
| d13 | Asarinin | 133-04-0 | 354.35 | 96.39 |
| d14 | Curcumin | 458-37-7 | 368.38 | 73.96 |
| d15 | Sesamin | 607-80-7 | 354.35 | 99.50 |
| d16 | Magnolin | 31008-18-1 | 416.46 | 65.08 |
| d17 | Demethoxycurcumin | 22608-11-3 | 338.35 | 99.33 |
| d18 | Schizandrin B | 61281-37-6 | 400.47 | 113.02 |
| d19 | Sesamolin | 526-07-8 | 370.35 | 81.63 |
| d20 | Schisantherin B | 58546-55-7 | 514.56 | 114.73 |
| d21 | Schisantherin A | 58546-56-8 | 536.57 | 117.73 |
| d22 | Gomisin A | 58546-54-6 | 416.47 | 112.44 |
| d23 | Fargesin | 31008-19-2 | 370.4 | 93.07 |
| d24 | Sesamol | 533-31-3 | 138.12 | 94.01 |
| Positive inhibitor | Tacrine | 321-64-2 | 198.26 | 10.96 |

1. **^1^H NMR spectrum and ^13^C NMR spectrum of compounds**


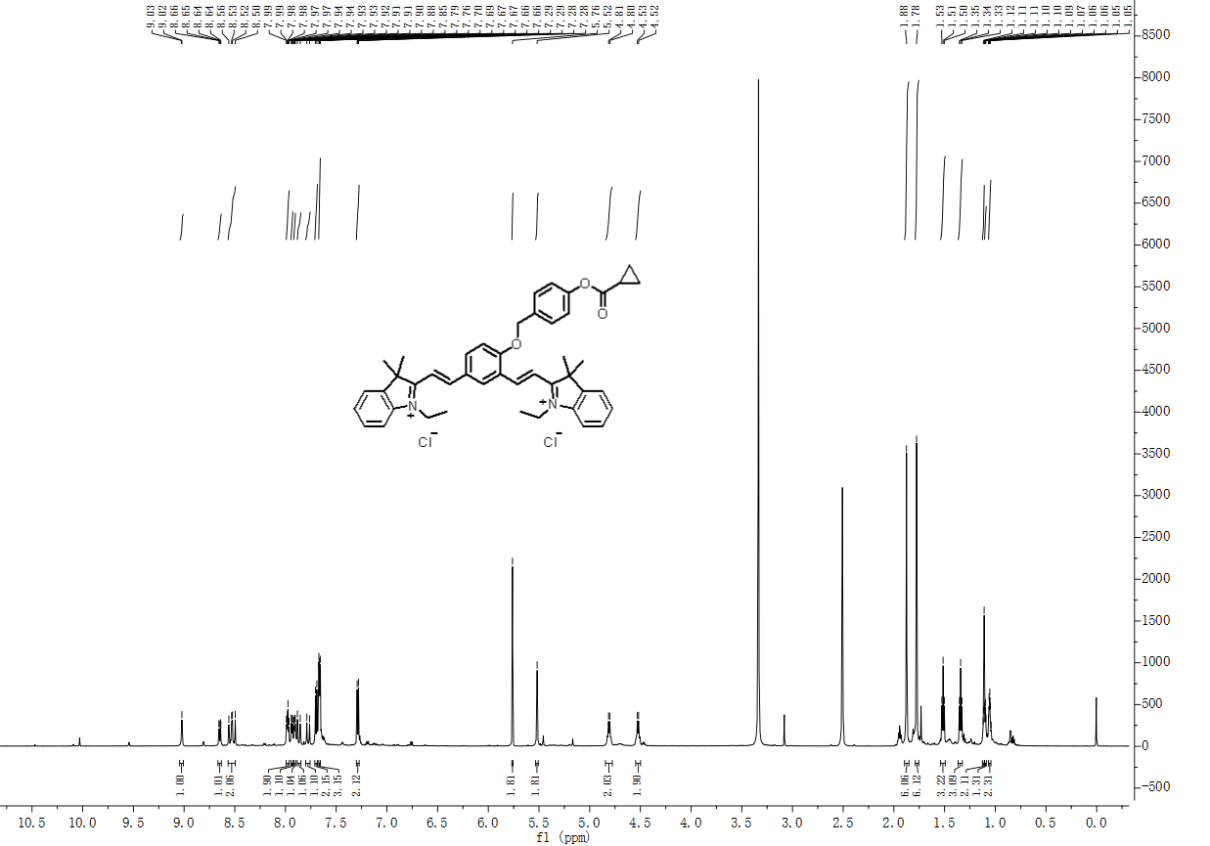


^1^H NMR spectrum of **CYBA** in DMSO-*d*6.


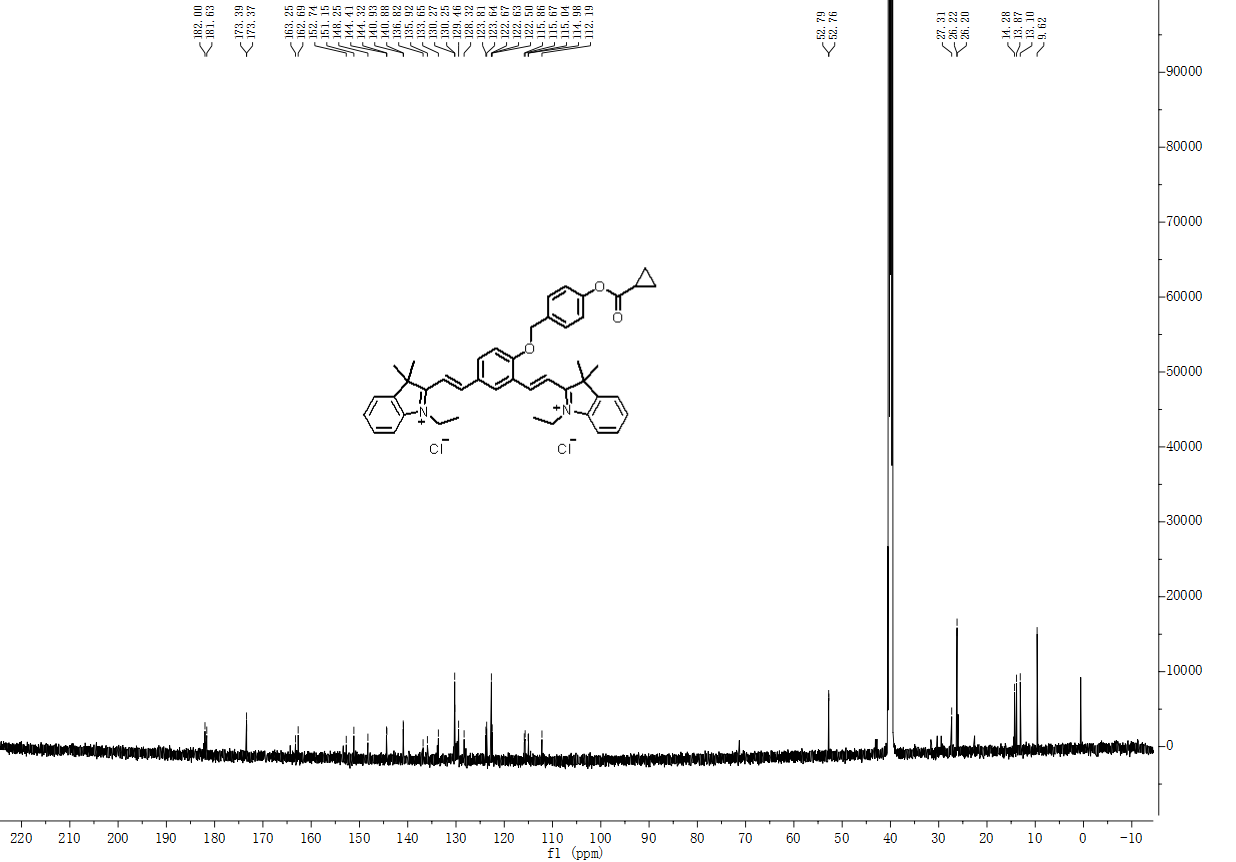


^13^C NMR spectrum of **CYBA** in DMSO-*d*6.


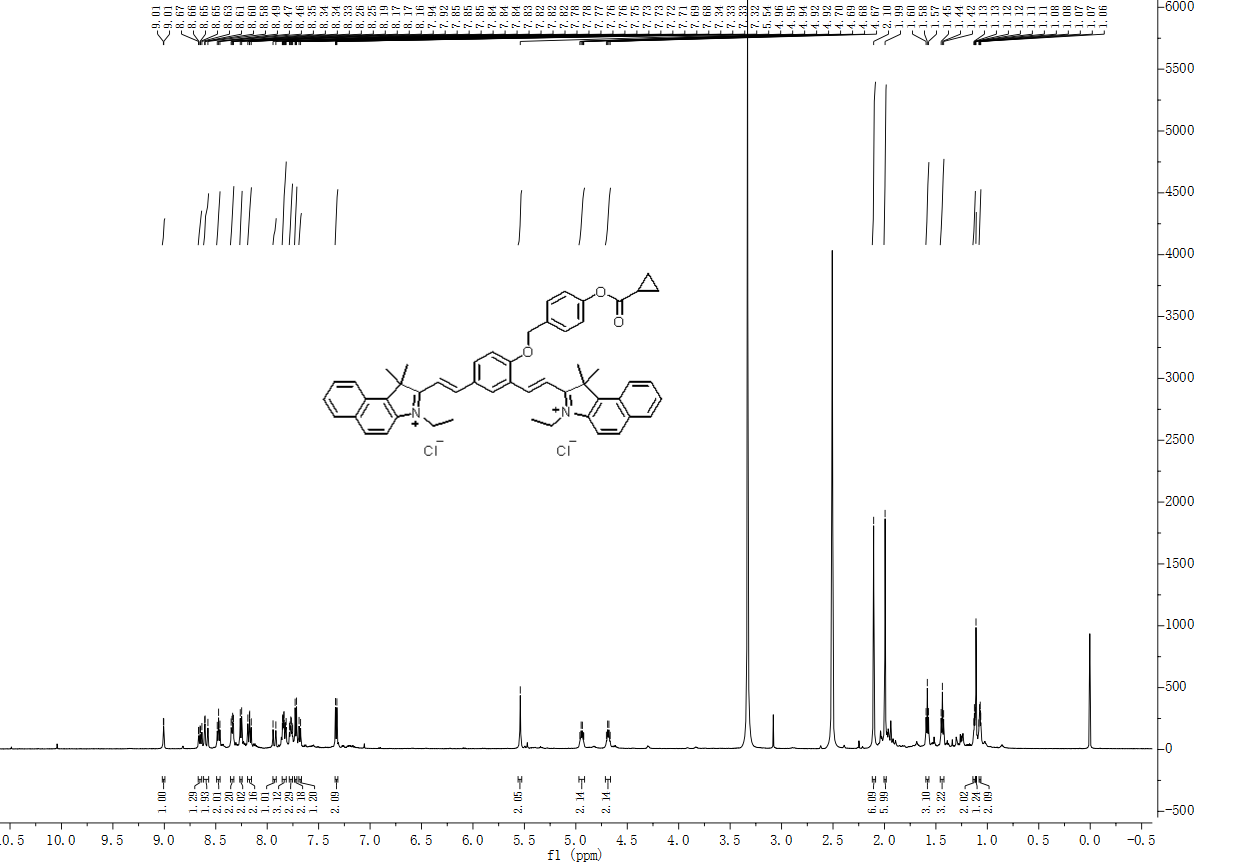


^1^H NMR spectrum of **CUBA** in DMSO-*d*6.


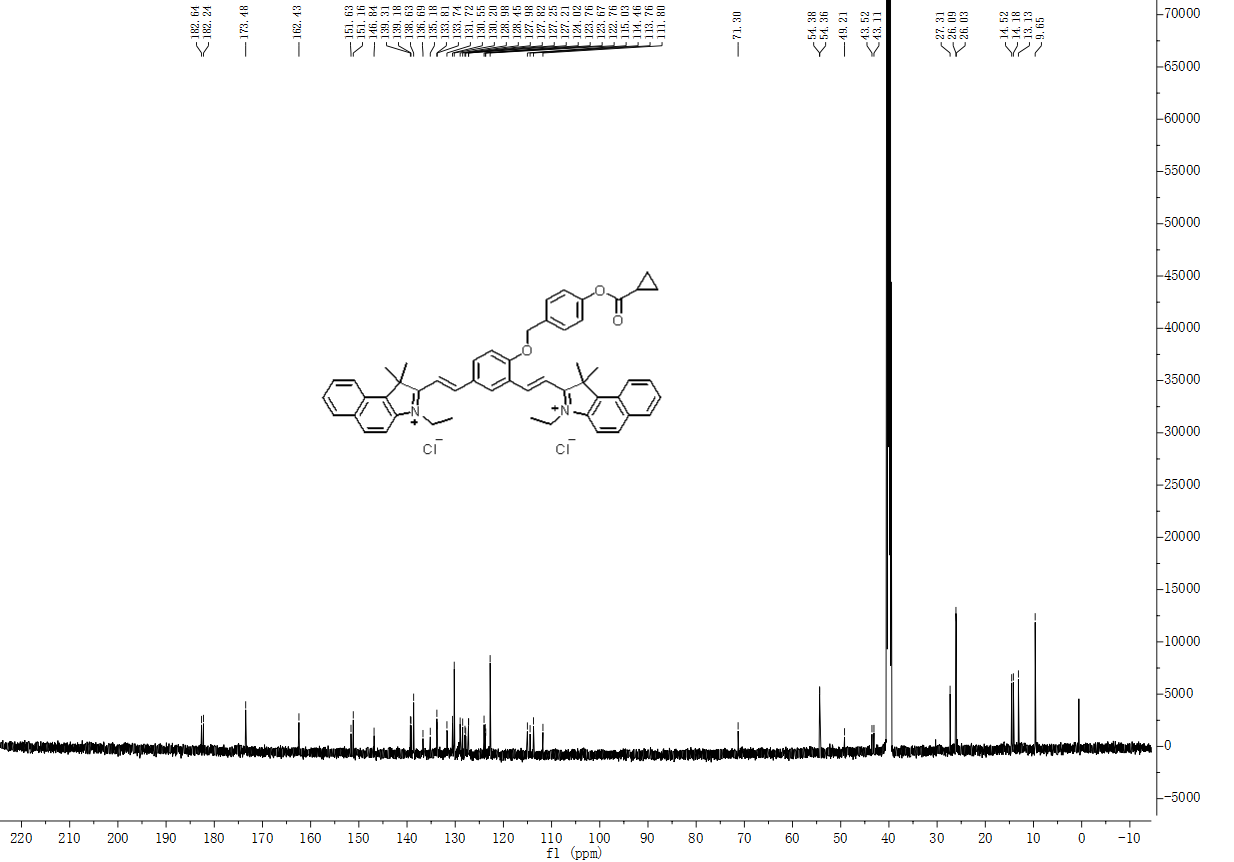


^13^C NMR spectrum of **CYBA** in DMSO-*d*6.


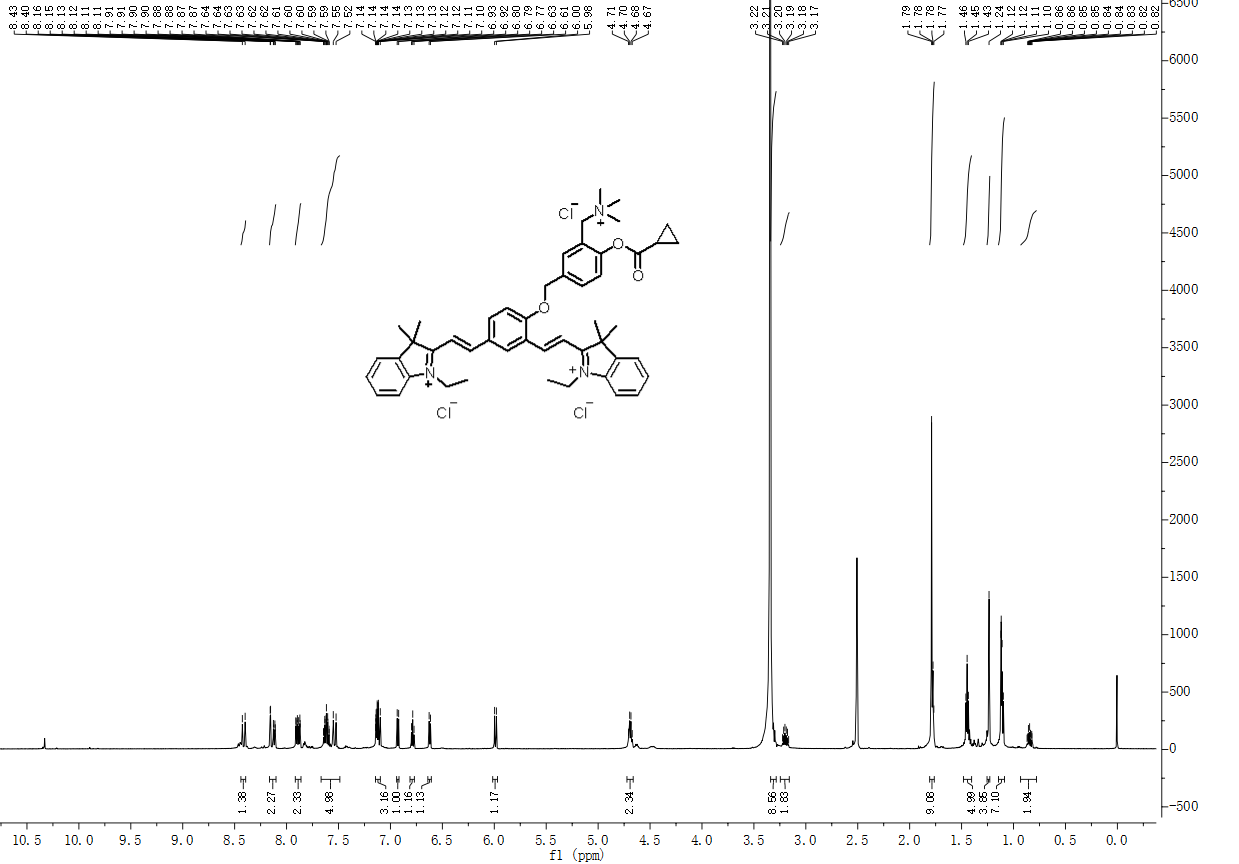


^1^H NMR spectrum of **CYNA** in DMSO-*d*6.


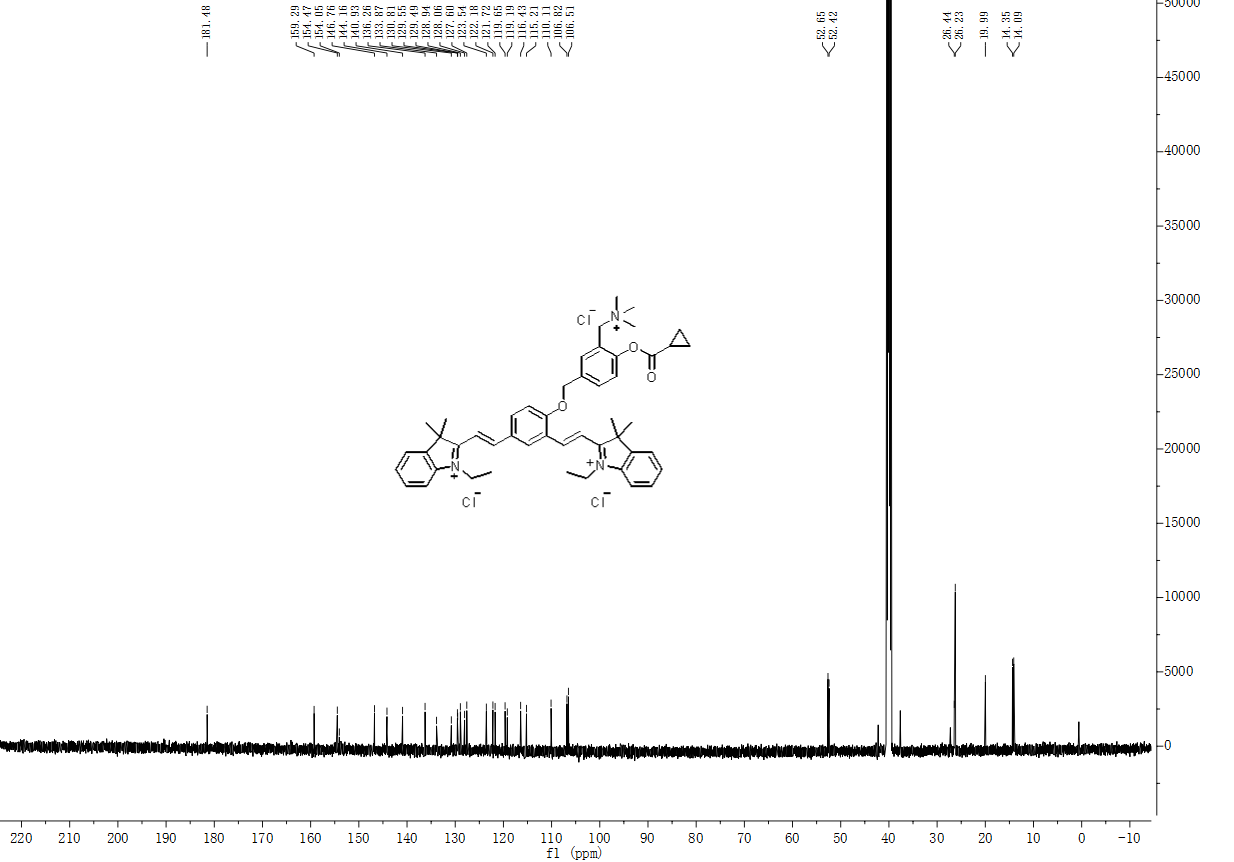


^13^C NMR spectrum of **CYNA** in DMSO-*d*6.


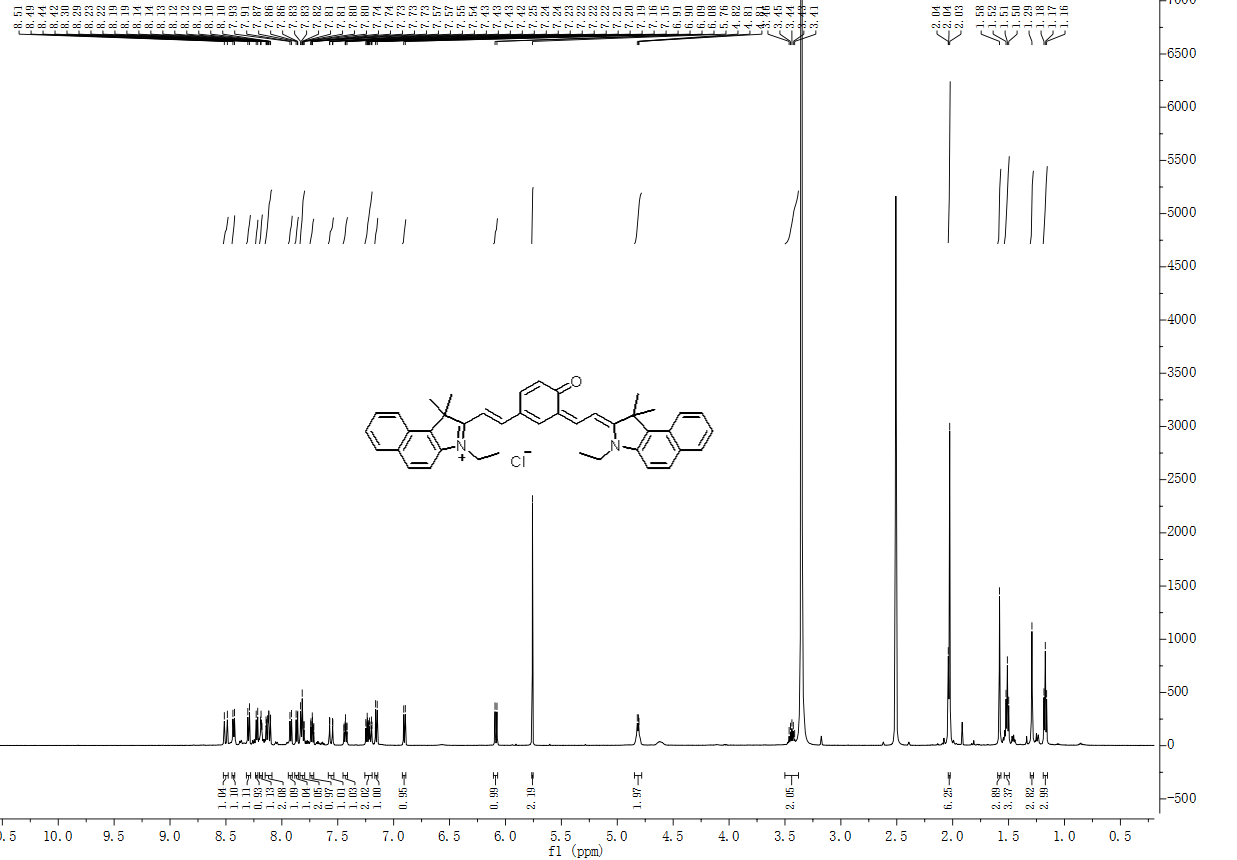


^1^H NMR spectrum of **TBBO** in DMSO-*d*6.


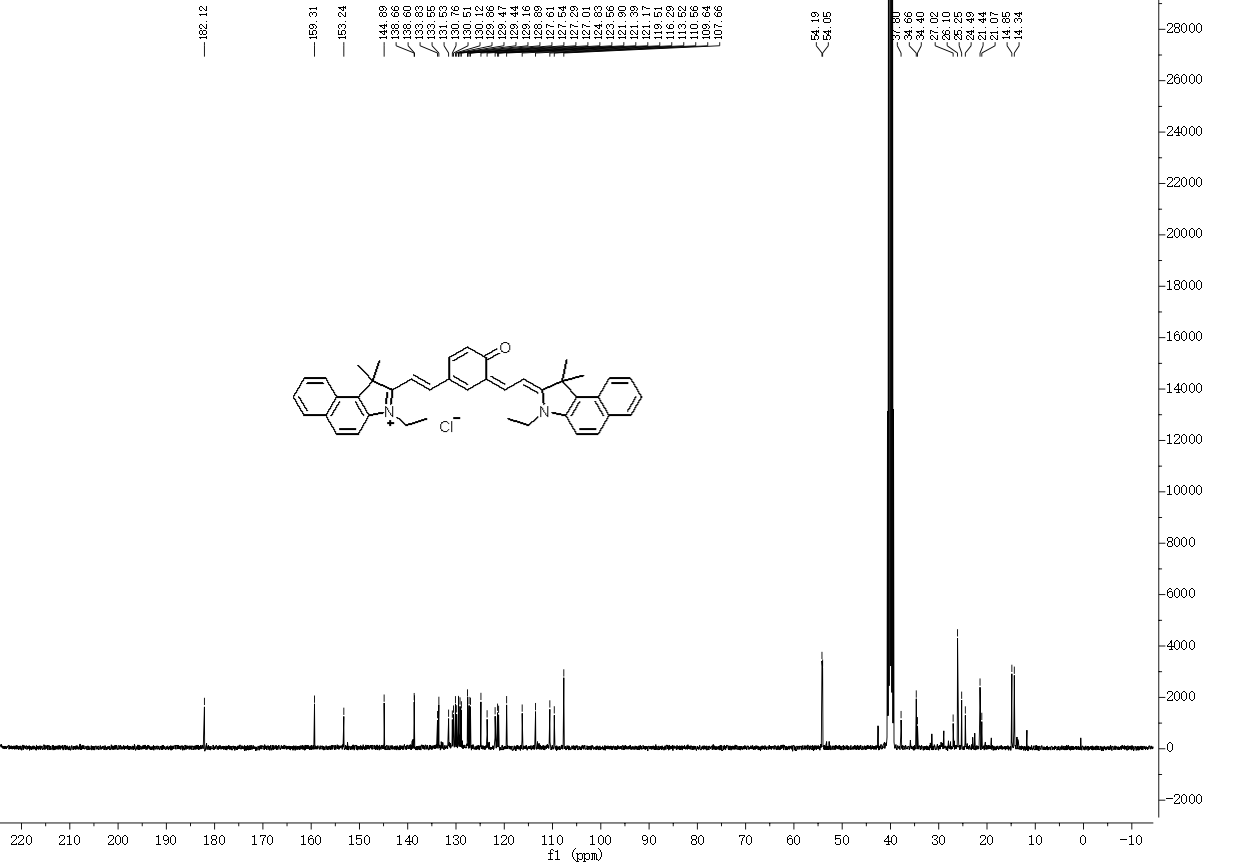


^13^C NMR spectrum of **TBBO** in DMSO-*d*6.


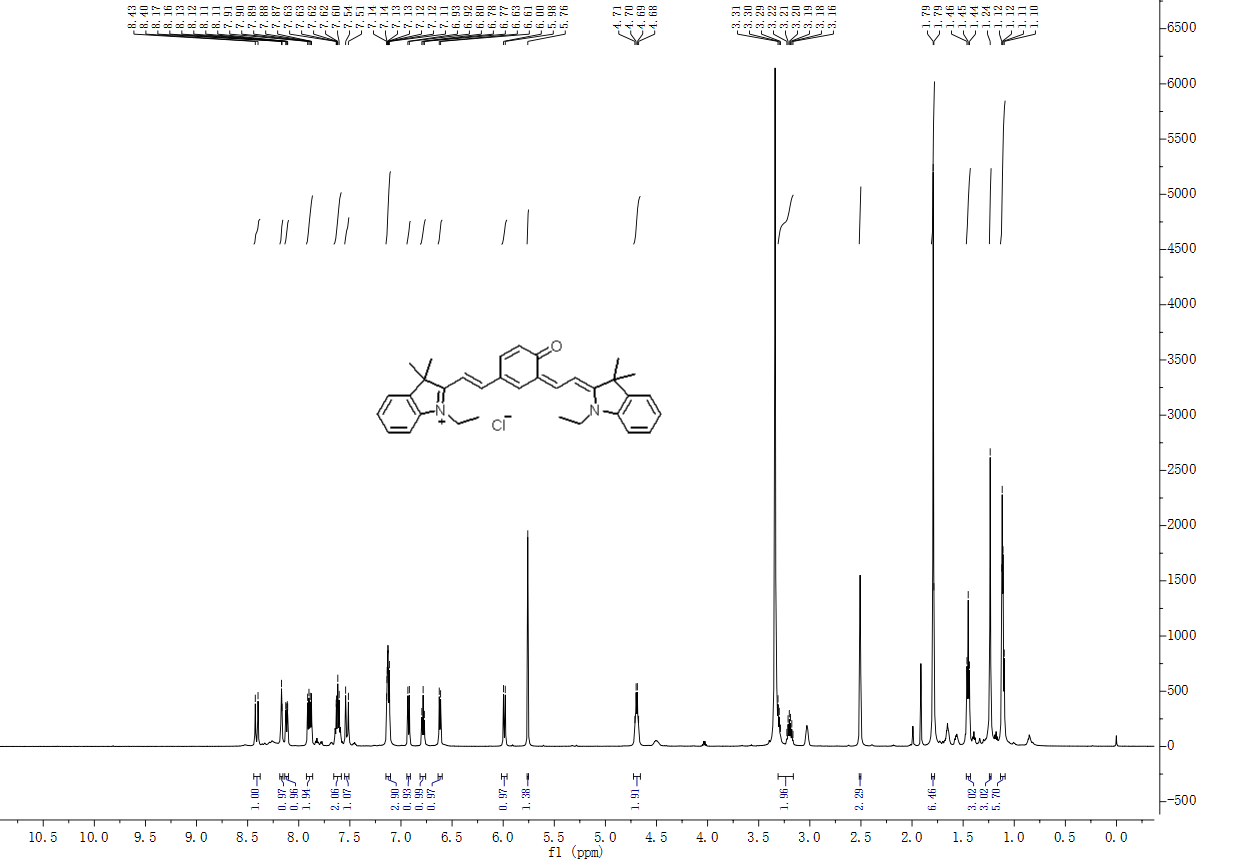


^1^H NMR spectrum of **TBO** in DMSO-*d*6.


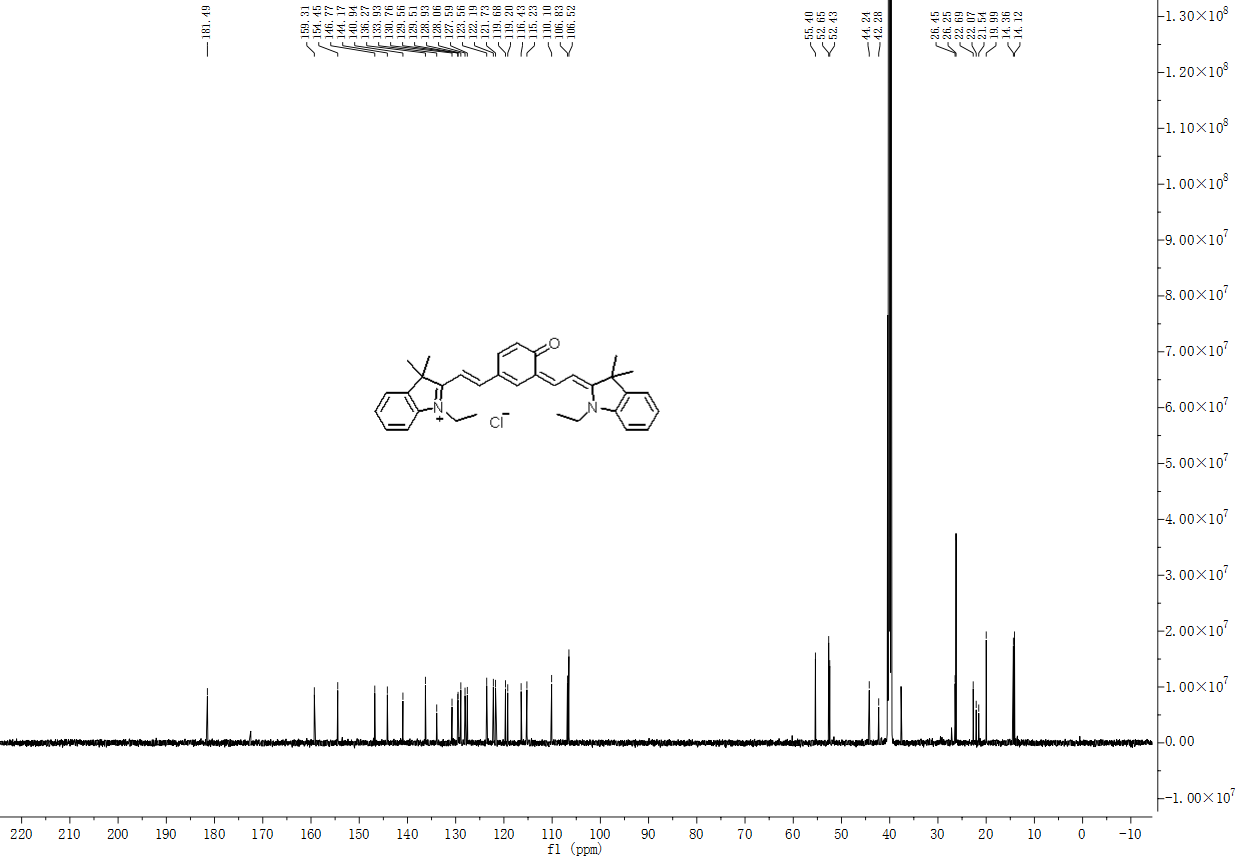


^13^C NMR spectrum of **TBO** in DMSO-*d*6.

**5. HRMS spectrum of compounds**


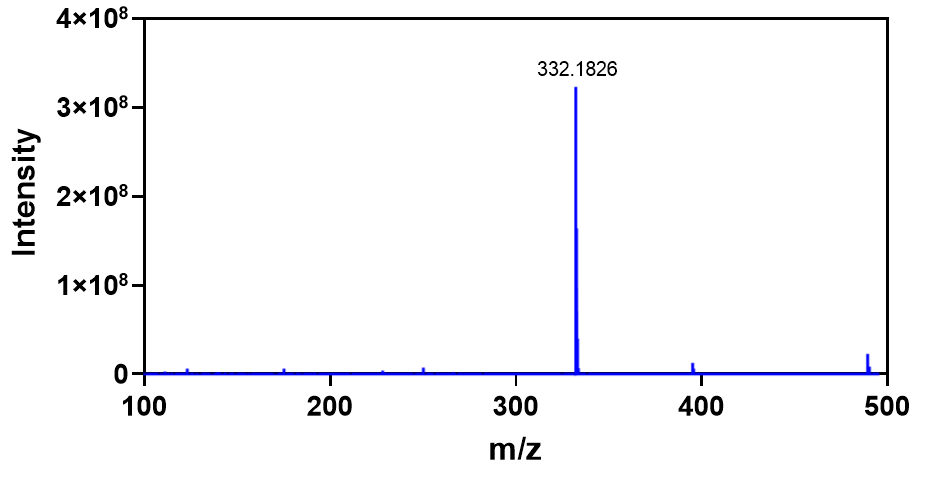


HRMS spectrum of **CYBA**.


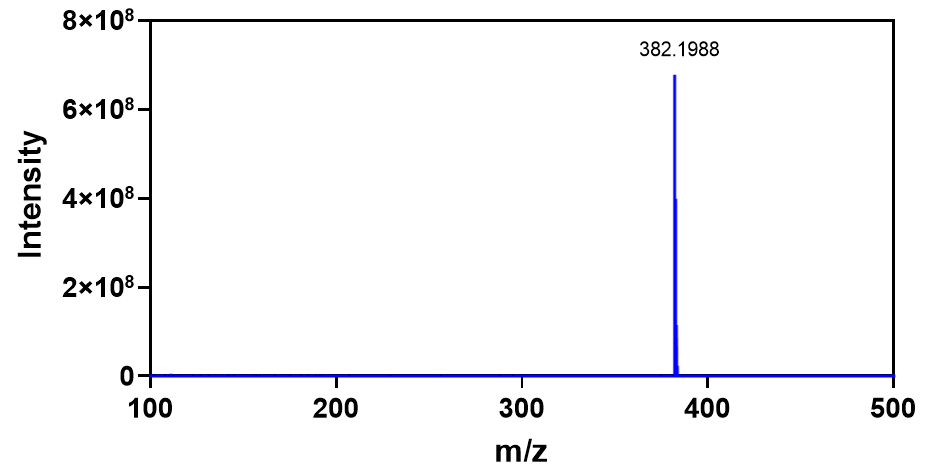


HRMS spectrum of **CUBA**.


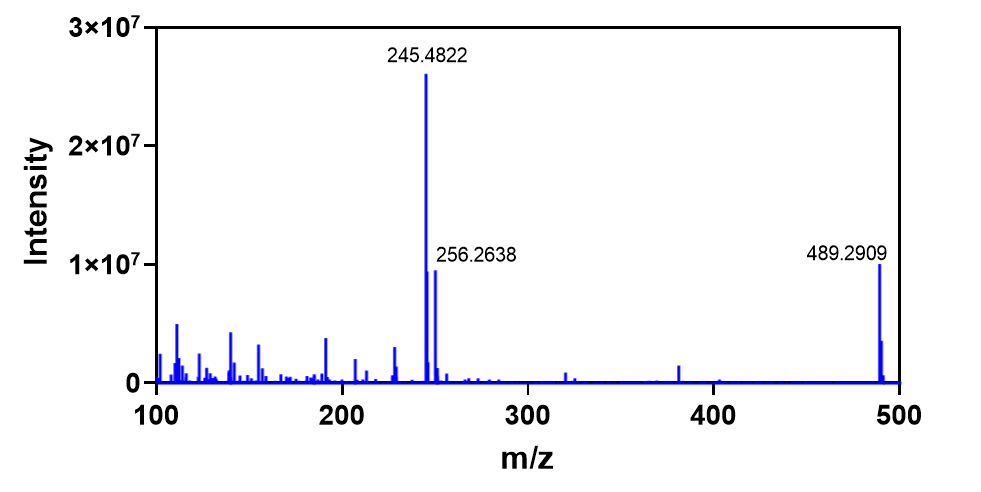


HRMS spectrum of **CYNA**.


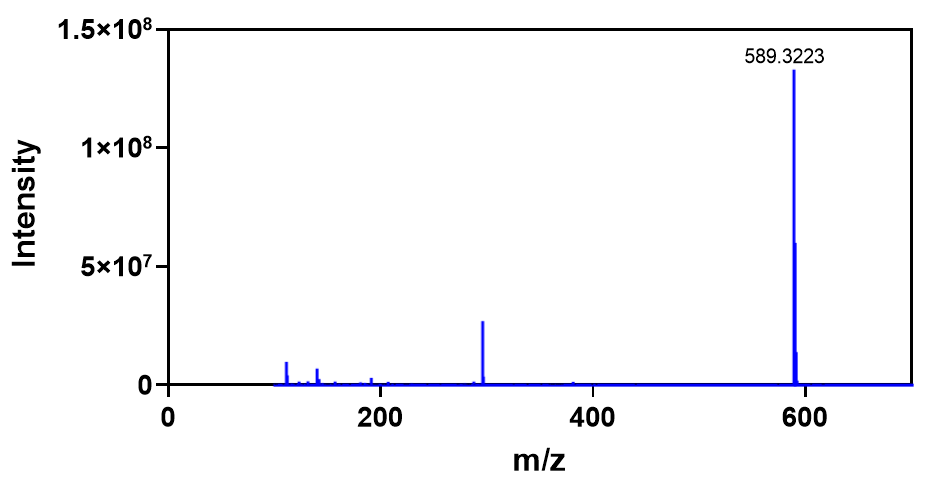


HRMS spectrum of **TBBO**.


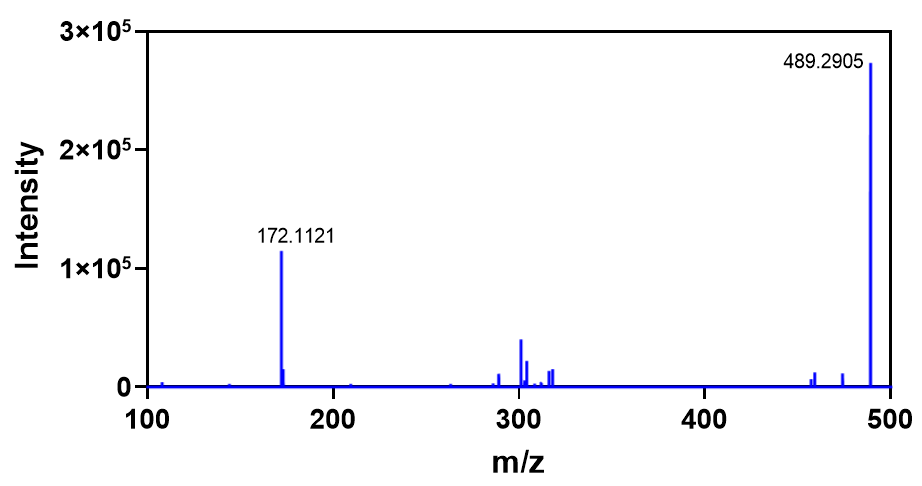


HRMS spectrum of **TBO.**
